# Model-Informed Translation of In Vitro Effects of Short-, Prolonged- and Continuous-Infusion Meropenem against *Pseudomonas aeruginosa* to Clinical Settings

**DOI:** 10.3390/antibiotics11081036

**Published:** 2022-08-01

**Authors:** Iris K. Minichmayr, Suzanne Kappetein, Margreke J. E. Brill, Lena E. Friberg

**Affiliations:** Department of Pharmacy, Uppsala University, P.O. Box 580, 75123 Uppsala, Sweden; iris.minichmayr@farmaci.uu.se (I.K.M.); Suzanne.kappetein@gmail.com (S.K.); margrekebrill@hotmail.com (M.J.E.B.)

**Keywords:** PKPD model, time-kill curve, prolonged infusion, continuous infusion, meropenem, beta-lactam, *Pseudomonas aeruginosa*, PK/PD index, pharmacometrics

## Abstract

Pharmacokinetic-pharmacodynamic (PKPD) models have met increasing interest as tools to identify potential efficacious antibiotic dosing regimens in vitro and in vivo. We sought to investigate the impact of diversely shaped clinical pharmacokinetic profiles of meropenem on the growth/killing patterns of *Pseudomonas aeruginosa* (ARU552, MIC = 16 mg/L) over time using a semi-mechanistic PKPD model and a PK/PD index-based approach. Bacterial growth/killing were driven by the PK profiles of six patient populations (infected adults, burns, critically ill, neurosurgery, obese patients) given varied pathogen features (e.g., EC_50_, growth rate, inoculum), patient characteristics (e.g., creatinine clearance), and ten dosing regimens (including two dose levels and 0.5-h, 3-h and continuous-infusion regimens). Conclusions regarding the most favourable dosing regimen depended on the assessment of (i) the total bacterial load or *f*T_>MIC_ (time that unbound concentrations exceed the minimum inhibitory concentration); (ii) the median or P_0.95_ profile of the population; and (iii) 8 h or 24 h time points. Continuous infusion plus loading dose as well as 3-h infusions (3-h infusions: e.g., for scenarios associated with low meropenem concentrations, P_0.95_ profiles, and MIC ≥ 16 mg/L) appeared superior to standard 0.5-h infusions at 24 h. The developed platform can serve to identify promising strategies of efficacious dosing for clinical trials.

## 1. Introduction

In an era of increasing antimicrobial resistance and a dwindling pipeline of novel antimicrobial drugs, the diligent use of the currently available antibiotics is more crucial than ever to preserve their activity. Rational dosing—balancing drug efficacy, safety and the emergence of resistance—poses a challenge particularly (i) for pathogens with reduced antibiotic susceptibility, including WHO critical priority pathogens like carbapenem-resistant *Pseudomonas aeruginosa*; and (ii) in special patient populations who frequently display variable pharmacokinetic processes like drug distribution or elimination [1,2]. In an effort to improve antibiotic exposure and therapeutic success for the abovementioned scenarios, alternative off-label dosing strategies have been proposed. As a prominent example, extended (prolonged or continuous) infusions of beta-lactams and other antibiotics with ‘time-dependent’ bactericidal killing have been increasingly popular owing to previous preclinical and clinical evidence. While the carbapenem antibiotic meropenem, for instance, is approved as 15–30 min infusions, 3-h infusions up to continuous infusions are commonly used [3,4]. Furthermore, renal function is an important factor to consider in meropenem dosing [3].

Clinical, randomised controlled trials are considered the most effective approach to support one therapy regimen for patients over another. For example, continuous infusion versus standard intermittent infusion is being investigated for carbapenems, inter alia in the BLING III study [5]. Although providing a high level of evidence, large clinical studies of this type (*n* = 7000 critically ill patients [5]) are resource-intensive and challenging, particularly if inclusion criteria are difficult to meet, as in the case of rare multidrug-resistant pathogens. These studies are usually restricted to highly-selected patient populations, types of infection and/or dosing regimens, leaving diverse other patient groups without explicit dosing recommendations. Furthermore, measures of clinical outcome of bacterial infections are usually restricted to indirect surrogates of bacterial eradication and disease severity determined at one specific time point (e.g., mortality or clinical cure after 90 days). Owing to these hurdles, preclinical studies that enable the direct quantification of the bacterial burden during antibiotic exposure, and translation of their results to humans, have been gaining increasing relevance in assessing antibiotic dosing strategies for patients [6].

Traditional approaches for preclinical-clinical translation and guidance on dosing regimens do not consider the time course of antibiotic exposure and response, but rather rely on summary measures like pharmacokinetic/pharmacodynamic (PK/PD) indices and targets [6]. The PK/PD index most closely associated with the efficacy of an antibiotic, e.g., *f*T_>MIC_ (cumulative time that unbound antibiotic concentrations exceed the minimum inhibitory concentration MIC) or *f*AUC/MIC (area under the unbound concentration-time curve/MIC), is typically determined in animals (e.g., murine infection) or in vitro time-kill experiments by determining the correlation between each index and the antibacterial response at one single time point (e.g., 24 h). The values of the PK/PD index, reflecting bacteriostasis, −1 log kill or −2 log kill at a certain time compared to start of antibiotic treatment, are referred to as PK/PD targets and constitute a common tool to select dosing regimens in humans (e.g., using probability of target attainment PTA analyses). However, this concept only allows for ‘black-and-white’ decisions (i.e., the PK/PD target is either met or not met given a certain dosing regimen) and lacks information on the development of antibiotic resistance [6,7]. Also, the MIC as the pharmacodynamic component in the PK/PD indices suffers from imprecision and represents bacterial susceptibility at merely one time point [8]. Furthermore, previous evidence has indicated that the magnitude of PK/PD targets can be influenced by different factors including the design of the underlying PKPD studies, MIC value, type of infection, species, patient population, half-life of the antibiotic, and shape of the pharmacokinetic profile [9]. For example, previous PKPD evidence on meropenem includes PK/PD targets of *f*T_>MIC_~20% (bacteriostasis in mice [10]), *f*T_>MIC_~35–55% (1−log10 reduction of *P. aeruginosa* [11]), *f*T_>MIC_~40–45% (bactericidal activity in mice [10]), T_>MIC_ ≥ 75% (febrile neutropenic patients [12], *f*T_>MIC_ = 100% (critically ill patients [13]), up to *f*C_min_/MIC > 5 (patients with lower respiratory tract infections [14]).

In contrast to the PK/PD index-based translational approach to inform human antibiotic dosing, model-based approaches allow for the consideration of the time course of bacterial dynamics [15]. Semi-mechanistic models are usually built upon data of in vitro time-kill experiments investigating bacterial growth, killing and resistance development of bacterial isolates (including rare and/or multi-drug resistant strains) during exposure to either constant, static antibiotic concentrations, or dynamic concentrations changing over time, e.g., mimicking pharmacokinetic profiles of patients. A comprehensive review has recently summarised such PK/PD models for time courses of antibiotic effects published since 1963, together with strategies to describe bacterial growth, regrowth, drug effects and interactions [16]. The developed models enable the prediction of continuous profiles of antibiotic concentrations (pharmacokinetics) and effects on the bacterial load (pharmacodynamics) over time, which were either previously measured in in vitro experiments, or represented yet untested scenarios. For instance, human pharmacokinetic profiles may be used to drive the outcome of the PKPD model and to explore in vitro-in vivo translation, i.e., the impact of pharmacokinetic profiles of specific patient populations on the bacteria given different dosing regimens. Hollow-fibre models have been increasingly used for this purpose, although also these experiments are usually limited to a few selected scenarios that often focus on the typical patient of a population of interest. Model-based approaches to characterise antibiotic PKPD have been widely used in research and are encouraged by regulatory agencies like the European Medicines Agency [17].

The current study aimed to investigate the impact of the shape of human pharmacokinetic (concentration-time) profiles of meropenem on the bacterial dynamics (growth/killing over time) of *P.* *aeruginosa* given diverse patient-, infection site-, and pathogen-related characteristics using a semi-mechanistic PKPD model versus a time-collapsed PK/PD index-based approach. We further sought to assess the relative benefit of different dosing strategies (e.g., short, prolonged and continuous infusions) expected for these clinically relevant scenarios to identify efficacious dosing regimens for special patient populations and specific clinical conditions.

## 2. Results

**Figure 1:**
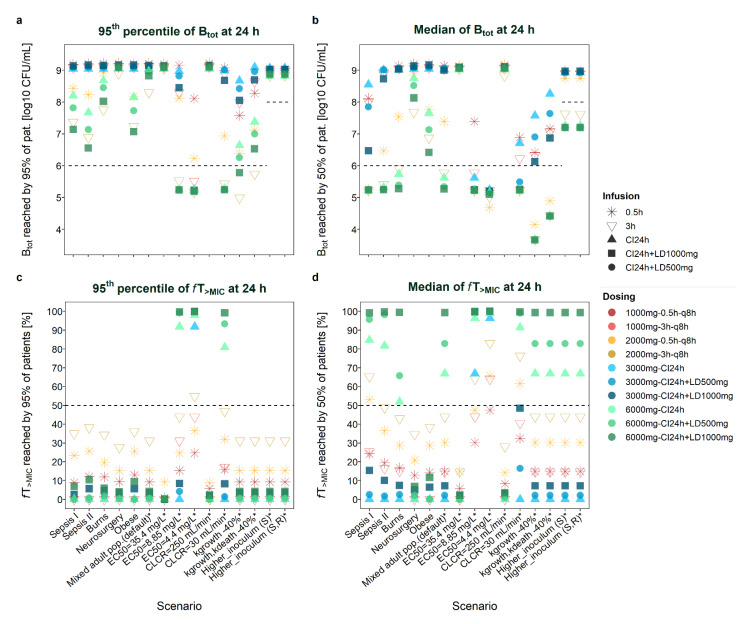
Total bacterial load (B_tot_, **a**,**b**) and *f*T_>MIC_ (time that meropenem concentrations exceed the minimum inhibitory concentration, Figures (**c**,**d**)) reached by 95% (**a**,**c**) or 50% (**b**,**d**) of the patient (pat.) population at 24 h after start of therapy given different scenarios. Sepsis I: Delattre et al. [23]; Sepsis II: Roberts et al. [22] (no renal dysfunction); EC_50_: drug concentration that produces 50% of E_max_ (maximum achievable kill rate constant); CLCR: creatinine clearance; kgrowth: rate constant of bacterial growth; kdeath: rate constant of natural bacterial death; higher inoculum: initial bacterial load of 8 log10 CFU/mL, either assumed to be susceptible or with an initial fraction in the resting state; scenarios marked with an asterisk * refer to a mixed adult population (default scenario; Li et al. [21]); CI: continuous infusion; LD: loading dose; q8h: every 8 h.

Of the ten dosage schemes investigated (e.g., high/low-dose short, prolonged- and continuous-infusion regimens ± loading dose), the regimen identified as the most favourable for a scenario (i.e., resulting in the lowest total bacterial load B_tot_ or highest *f*T_>MIC_) differed depending on (i) whether the median or the P_0.95_ profile of the population was assessed; (ii) the time of assessment (24 h or 8 h); and (iii) on the evaluated measure of efficacy (B_tot_ or *f*T_>MIC_). Comparisons of total bacterial load and *f*T_>MIC_ given the different populations (Table S1) dosing regimens, scenarios, times of assessment (24 h/8 h) and profiles (median/P_0.95_) are presented graphically (Figure 1: B_tot_ and *f*T_>MIC_ at 24 h; Figures S1 and S2: time courses of meropenem concentrations and bacterial load; Figure S3: B_tot_ and *f*T_>MIC_ at 8 h) and in tables (Table 1: B_tot_P0.95_ at 8 h and 24 h; Table 2: *f*T_>MIC_P0.95_ at 8 h and 24 h; Table S2: B_tot_median_ at 8 h and 24 h; Table S3: *f*T_>MIC_median_ at 8 h and 24 h). Table 3 highlights the most favourable dosing regimens with respect to total bacterial load and *f*T_>MIC_ (at 8 h and 24 h) based on plasma PKPD profiles (covering 95% of patients) for the diverse investigated scenarios.

Of the six diverse patient populations used to inform the pharmacokinetic component of the PKPD model (Table S1), the median representative of neurosurgery patients [18] exhibited the lowest meropenem concentrations given continuous infusion, followed by the median obese patient [19], burns patient [20], and the median profile of a mixed infected population (default scenario [21]). Critically ill patients exhibited the highest trough concentrations (to be precise, the septic population without renal dysfunction [22] displayed lower concentrations than the more diverse septic population [23]). When assuming CLCR values of 30 mL/min and 250 mL/min (instead of the default value of 83 mL/min) for the general mixed population (Li et al. [21]), overall highest and lowest concentration-time profiles were achieved compared to all other median population profiles. Differences in the pharmacokinetic (concentration-time) profile were naturally reflected by different shapes of the pharmacodynamic (bacterial load-time) profile and by different *f*T_>MIC_ values. The following results focus on the resistant ARU552 strain unless stated otherwise.

**Table 1:** Total bacterial load at 8 h and 24 h based on PKPD profiles covering 95% of patients.

				Total Bacterial Load (B_tot_, P_0.95_ Profile)									
				TDD ~3000 mg/Day					TDD ~6000 mg/Day				
Model	Population	Scenario	B_tot_ Time	II 1000 mg q8h		CI 3000 mg/24h			II 2000 mg q8h		CI 6000 mg/24 h		
				0.5 h	3 h	No LD	LD 500	LD 1000	0.5 h	3 h	No LD	LD 500	LD 1000
Li 2006 [21]	Infected adults	Default (Plasma, CLCR 83 mL/min, MIC 16 mg/L)	8 h	7.66	7.80	8.07	7.77	7.36	7.27	6.81	7.46	7.08	6.64
			24 h	9.21	9.11	9.04	9.10	9.15	9.15	8.30	8.97	8.95	8.82
Doh 2010 [20]	Burns	Plasma	8 h	7.54	7.69	8.03	7.67	7.18	7.08	6.63	7.26	6.81	6.28
			24 h	9.21	9.10	9.04	9.10	9.15	8.91	7.75	8.68	8.45	8.02
Delattre 2012 [23]	Sepsis	Plasma	8 h	7.58	7.74	8.02	7.67	7.08	6.84	6.56	7.23	6.75	6.07
			24 h	9.19	9.08	9.04	9.09	9.14	8.43	7.37	8.20	7.82	7.14
		Lung	8 h	8.15	8.16	8.18	8.16	8.12	7.98	8.06	8.14	8.12	8.05
			24 h	9.10	9.05	9.05	9.05	9.10	9.19	9.05	9.05	9.05	9.08
Roberts 2009 [22]	Sepsis	Plasma	8 h	7.43	7.57	7.95	7.50	6.85	6.76	6.32	6.89	6.33	**5.66**
			24 h	9.20	9.10	9.04	9.10	9.16	8.23	6.89	7.67	7.14	6.56
		Lung	8 h	8.12	8.16	8.17	8.15	8.08	7.85	8.01	8.13	8.09	7.99
			24 h	9.07	9.05	9.05	9.05	9.07	9.20	9.06	9.05	9.05	9.08
Lu 2016 [18]	Neurosurgery	Plasma	8 h	7.70	7.91	8.11	7.89	7.54	7.34	7.07	7.74	7.47	7.08
			24 h	9.21	9.11	9.05	9.10	9.16	9.20	8.89	9.04	9.07	9.10
		Cerebrospinal fluid	8 h	8.18	8.18	8.18	8.18	8.18	8.18	8.18	8.18	8.18	8.18
			24 h	9.05	9.05	9.05	9.05	9.05	9.05	9.05	9.05	9.05	9.05
Wittau 2015 [19]	Obese	Plasma	8 h	7.46	7.64	7.99	7.60	6.94	6.75	6.44	7.07	6.57	**5.86**
			24 h	9.20	9.09	9.04	9.09	9.16	8.18	7.24	8.15	7.73	7.07
		s.c. interstitial space fluid	8 h	7.74	7.92	8.10	7.91	7.46	7.07	7.04	7.64	7.37	6.83
			24 h	9.17	9.04	9.04	9.07	9.13	8.92	8.79	9.01	9.02	8.99
		Peritoneal fluid	8 h	7.49	7.67	8.00	7.65	7.01	6.79	6.52	7.16	6.69	**6.00**
			24 h	8.52	8.42	8.76	8.60	8.47	8.08	7.23	7.47	7.23	6.95
Li 2006 [21]	Infected adults	CLCR 30 mL/min	8 h	7.21	7.21	7.72	6.97	6.13	6.37	**5.76**	6.12	**5.35**	**4.63**
			24 h	9.08	8.91	8.99	9.01	8.68	6.94	**5.44**	**5.28**	**5.25**	**5.25**
		CLCR 250 mL/min	8 h	7.98	8.09	8.16	8.06	7.84	7.68	7.67	8.05	7.93	7.69
			24 h	9.21	9.10	9.05	9.09	9.15	9.22	9.14	9.04	9.07	9.11
		EC_50_ 4.4 mg/L	8 h	6.74	**5.75**	**5.37**	**4.39**	**4.15**	6.09	**5.01**	**3.96**	**3.89**	**3.86**
			24 h	8.11	**5.52**	**5.25**	**5.24**	**5.23**	6.23	**5.18**	**5.23**	**5.22**	**5.20**
		EC_50_ 8.8 mg/L	8 h	7.27	6.81	7.46	6.64	6.06	6.74	**5.75**	**5.39**	**4.72**	**4.40**
			24 h	9.15	8.30	8.97	8.82	8.45	8.12	**5.53**	**5.25**	**5.25**	**5.24**
		EC_50_ 35.4 mg/L	8 h	7.99	8.12	8.17	8.11	7.93	7.66	7.80	8.07	7.99	7.77
			24 h	9.18	9.05	9.05	9.07	9.14	9.21	9.11	9.04	9.06	9.10
		k_growth_, k_death_ −40%	8 h	6.78	6.93	7.20	6.90	6.48	6.39	**5.93**	6.58	6.20	**5.76**
			24 h	8.28	8.64	9.09	8.96	8.70	7.12	**5.74**	7.38	7.00	6.53
		k_growth_ −40%	8 h	6.53	6.68	6.95	6.65	6.23	6.14	**5.68**	6.33	**5.95**	**5.51**
			24 h	7.58	7.99	8.67	8.42	8.05	6.38	**4.99**	6.64	6.26	**5.78**
		Higher initial bact. load (S)	8 h	9.17	9.19	9.22	9.17	9.03	9.00	8.66	8.96	8.80	8.50
			24 h	9.05	9.02	9.06	9.04	9.02	8.97	8.82	8.82	8.84	8.88
		Higher initial bact. load (SR)	8 h	9.16	9.19	9.22	9.17	9.02	8.99	8.65	8.95	8.79	8.49
			24 h	9.04	9.02	9.06	9.04	9.02	8.97	8.83	8.82	8.84	8.88

Abbreviations: TDD: total daily dose; II: intermittent dosing; CI: continuous infusion; q8/24h: every 8/24 hours; LD: loading dose (in mg); CLCR: creatinine clearance; MIC: minimum inhibitory concentration; s.c. subcutaneous; EC_50_: drug concentration (mg/L) that produces 50% of maximum achievable kill rate constant; k_growth_: rate constant of bacterial growth; k_death_: rate constant of bacterial death; higher initial bacterial load: 8 log10 CFU/mL, either assumed to be susceptible (S) or with an initial fraction in the resting state (SR); categories/colour code (scenarios with initial bacterial load 6 log10 CFU/mL): bacteriostasis/net bacterial kill (bold values): >5–6 log10 CFU/mL (beige), >4–5 log10 CFU/mL (light cyan), ≤4 log10 CFU/mL (mint); net bacterial growth (non-bold values): >6–7 log10 CFU/mL (light red), >7–8 log10 CFU/mL (lilac), >8 log10 CFU/mL (violet red). Colours were intended to be accessible to people who are colour-blind (https://davidmathlogic.com/colorblind; access date 26 July 2022).

**Table 2:** *f*T_>MIC_ at 8 h and 24 h after start of therapy based on PKPD profiles covering 95% of patients.

				*f*T_>MIC_ (P_0.95_ Profile)									
				TDD ~3000 mg/Day					TDD ~6000 mg/Day				
Model	Population	Scenario	*f*T_>MIC_ time	II 1000 mg q8h		CI 3000 mg/24h			II 2000 mg q8h		CI 6000 mg/24 h		
				0.5 h	3 h	No LD	LD 500	LD 1000	0.5 h	3 h	No LD	LD 500	LD 1000
Li 2006 [21]	Infected adults	Default (Plasma, CLCR 83 mL/min, MIC 16 mg/L)	8 h	9.2	0	0	0.6	10.4	15.2	*30.4*	0	1.9	12.7
			24 h	9.2	0	0	0.2	3.5	15.4	*31.2*	0	0.7	4.2
Doh 2010 [20]	Burns	Plasma	8 h	11.9	0	0	4.4	14.2	19.6	*33.8*	0	5.8	18.1
			24 h	12.0	0	0	1.5	4.7	19.7	*34.4*	0	1.9	6.0
Delattre 2012 [23]	Sepsis	Plasma	8 h	2.1	0	0	0	6.9	*22.9*	*32.7*	0	0	19.2
			24 h	9.0	0	0	0	2.6	*23.3*	*25.0*	0	0	6.9
		Lung	8 h	0	0	0	0	0	0	0	0	0	0
			24 h	0	0	0	0	0	0	0	0	0	0
Roberts 2009 [22]	Sepsis	Plasma	8 h	11.5	0	0	0	17.1	*25.6*	*37.7*	0	1.9	*31.2*
			24 h	12.2	0	0	0	5.7	*25.6*	*38.1*	0	0.7	10.5
		Lung	8 h	0	0	0	0	0	0.8	0	0	0	0
			24 h	0	0	0	0	0	2.9	0	0	0	0
Lu 2016 [18]	Neurosurgery	Plasma	8 h	9.4	0	0	1	10.6	15.2	*27.5*	0	1.2	12.3
			24 h	9.5	0	0	0.3	3.5	15.3	*27.6*	0	0.4	4.1
		Cerebrospinal fluid	8 h	0	0	0	0	0	0	0	0	0	0
			24 h	0	0	0	0	0	0	0	0	0	0
Wittau 2015 [19]	Obese	Plasma	8 h	12.9	0	0	0	17.1	*25.4*	*35.6*	0	0	*28.1*
			24 h	13.0	0	0	0	5.7	*25.6*	*36.1*	0	0	9.4
		s.c. interstitial space fluid	8 h	1.9	0	0	0	2.5	6.7	7.3	0	0	3.5
			24 h	6.0	0	0	0	2.5	*20.1*	*22.5*	0	0	3.5
		Peritoneal fluid	8 h	4.0	0	0	0	5.3	8.3	11.4	0	0	8.4
			24 h	12.2	0	0	0	5.3	*24.9*	*34.7*	0	0	8.4
Li 2006 [21]	Infected adults	CLCR 30 mL/min	8 h	*29.0*	*34.4*	0	*34.0*	**98.3**	**58.5**	**70.9**	**74.4**	**97.3**	**99.0**
			24 h	16.0	17.2	0	1.5	8.3	*32.0*	**46.9**	**80.9**	**93.4**	**99.2**
		CLCR 250 mL/min	8 h	5.8	0	0	0	6.2	8.8	0	0	0	6.5
			24 h	5.8	0	0	0	2.1	8.8	0	0	0	2.2
		EC_50_ 4.4 mg/L	8 h	*24.4*	**43.8**	**75.6**	**99.2**	**99.8**	*35.8*	**54.6**	**94.0**	**99.2**	**99.8**
			24 h	*24.6*	**43.9**	**91.9**	**99.7**	**99.9**	*36.5*	**54.8**	**98.0**	**99.7**	**99.9**
		EC_50_ 8.8 mg/L	8 h	15.2	*30.4*	0	15.7	*25.4*	*24.4*	**43.8**	**75.6**	**97.7**	**99.2**
			24 h	15.4	*31.2*	0	4.2	8.5	*24.5*	**43.9**	**91.9**	**99.2**	**99.7**
		EC_50_ 35.4 mg/L	8 h	0.2	0	0	0	0.4	9.2	0	0	0	0.6
			24 h	0.8	0	0	0	0.1	9.2	0	0	0	0.2
		k_growth_, k_death_ −40%	8 h	9.2	0	0	0.6	10.4	15.2	*30.4*	0	1.9	12.7
			24 h	9.2	0	0	0.2	3.5	15.4	*31.2*	0	0.7	4.2
		k_growth_ −40%	8 h	9.2	0	0	0.6	10.4	15.2	*30.4*	0	1.9	12.7
			24 h	9.2	0	0	0.2	3.5	15.4	*31.2*	0	0.7	4.2
		Higher initial bact. load (S)	8 h	9.2	0	0	0.6	10.4	15.2	30.4	0	1.9	12.7
			24 h	9.2	0	0	0.2	3.5	15.4	31.2	0	0.7	4.2
		Higher initial bact. load (SR)	8 h	9.2	0	0	0.6	10.4	15.2	30.4	0	1.9	12.7
			24 h	9.2	0	0	0.2	3.5	15.4	31.2	0	0.7	4.2

Abbreviations: TDD: total daily dose; II: intermittent dosing; CI: continuous infusion; q8/24h: every 8/24 hours; LD: loading dose (in mg); CLCR: creatinine clearance; MIC: minimum inhibitory concentration; s.c. subcutaneous; EC_50_: drug concentration (mg/L) that produces 50% of maximum achievable kill rate constant; k_growth_: rate constant of bacterial growth; k_death_: rate constant of natural bacterial death; higher initial bacterial load: 8 log10 CFU/mL, either assumed to be susceptible (S) or with an initial fraction in the resting state (SR); categories/colour code: *f*T_>MIC_ = 95–100% (bold/underlined, mint), *f*T_>MIC_ = 40–<95% (bold, light cyan), *f*T_>MIC_ = 20–<40% (italic, beige), *f*T_>MIC_ = 10–<20% (non-bold/non-italic, light red), *f*T_>MIC_ = <10% (non-bold/non-italic, violet red). Colours were intended to be accessible to people who are colour-blind (https://davidmathlogic.com/colorblind; access date 26 July 2022).

**Table 3:** Most favourable dosing regimens (total daily dose ≥6000 mg) with respect to total bacterial load and *f*T_>MIC_ at 8 h and 24 h based on plasma PKPD profiles (covering 95% of patients) for the investigated scenarios.

Time	0.5 h	3 h	CI	CI + LD_500mg_	CI + LD_1000mg_
24 h		Infected adults (8.30/31.2)			
8 h		Infected adults (30.4), *			Infected adults (6.64)
24 h		Burns (7.75/34.4)			*
8 h		Burns (33.8)			Burns (6.28)
24 h		Sepsis I (25.0), *			Sepsis I (7.14)
8 h		Sepsis I (32.7)			Sepsis I (6.07)
24 h		Sepsis II (38.1)			Sepsis II (6.56)
8 h		Sepsis II (37.7)			Sepsis II (5.66)
24 h	*	Neurosurgery (8.89/27.6)	*	*	*
8 h	*	Neurosurgery (7.07/27.5)			*
24 h		Obese (36.1), *			Obese (7.07)
8 h		Obese (35.6)			Obese (5.86)
24 h		*	*	CLCL 30 mL/min (5.25)	CLCL 30 mL/min (5.25/99.2)
8 h				^	CLCL 30 mL/min (4.63/99.0)
24 h	CLCR 250 mL/min (8.8), *	*	CLCR 250 mL/min (9.04)	*	*
8 h	CLCR 250 mL/min (8.8), *	CLCR 250 mL/min (7.67)	*	*	*
24 h		EC_50_ 4.4 mg/L (5.18)	*	*, ^	EC_50_ 4.4 mg/L (99.9), *
8 h			*, ^	*, ^	EC_50_ 4.4 mg/L (3.86/99.8), ^
24 h			*	*, ^	EC_50_ 8.8 mg/L (5.24/99.7)
8 h				^	EC_50_ 8.8 mg/L (4.40/99.2)
24 h	EC_50_ 35.4 mg/L (9.2), *	*	EC_50_ 35.4 mg/L (9.04)	*	*
8 h	EC_50_ 35.4 mg/L (7.66/9.2)	*		*	*
24 h		k_growth,death_ −40% (5.74/31.2)			
8 h		k_growth,death_ −40% (30.4), *			k_growth,death_ −40% (5.76)
24 h		k_growth_ −40% (4.99/31.2)			
8 h		k_growth,death_ −40% (30.4), *			k_growth_ −40% (5.51)
24 h	*	Higher BL (S) (8.82/31.2)	Higher BL (S) (8.82)	*	*
8 h		Higher BL (S) (30.4), *		*	Higher BL (S) (8.50)
24 h	*	Higher BL (SR) (31.2), *	Higher BL (SR) (8.82)	*	*
8 h		Higher BL (SR) (30.4), *		*	Higher BL (SR) (8.49)

Values in brackets represent lowest total bacterial load (log10 CFU/mL) or, if underlined, highest *f*T_>MIC_ for each scenario. * dosing regimens leading to a total bacterial load that deviates < 5% from the most favourable dosing regimen; ^ dosing regimens leading to a *f*T_>MIC_ value that deviates < 5% from the most favourable dosing regimen. Abbreviations: CI: continuous infusion; LD: loading dose (followed by continuous infusion of 6000 mg/24 h); CLCR: creatinine clearance; EC_50_: drug concentration (mg/L) that produces 50% of maximum achievable kill rate; kgrowth: rate constant of bacterial growth; kdeath: rate constant of bacterial death; higher initial bacterial load (BL): 8 log10 CFU/mL, either assumed to be susceptible (S) or with an initial fraction in the resting state (SR).

### 2.1. Evaluation of Total Bacterial Load

#### 2.1.1. Patient-Related Characteristics and Site of Infection

Given the typical patient characteristics of the six populations, the highest bacterial load was observed for a general mixed population [21] with CLCR = 250 mL/min and for neurosurgery patients [18], whereas lowest bacterial counts were observed for a general mixed population with CLCR = 30 mL/min and for critically ill patients [23]. Given the default MIC of the resistant strain (MIC = 16 mg/L), 3-h infusions generally performed better than (or comparably well as) standard 0.5-h infusions with respect to bacterial reduction at 24 h (see Figure S2 for all six populations).

At 8 h after start of dosing (i.e., after one dose in case of intermittent dosing), bacterial reduction by a 3-h infusion was frequently still below that achieved by a 0.5-h infusion, although this trend was reversed at 24 h (see above).

##### Most Favourable Regimens

The *continuous-infusion regimen with highest loading dose* (CI+LD_1000mg_) resulted in the lowest bacterial load (or in bacterial counts comparable to after 3-h infusions) at 24 h for most scenarios, both when assessing the median bacterial load value (B_tot_median_24h_) or the 95th percentile of B_tot_ values (B_tot_P0.95_24h_). For example, B_tot_P0.95_24h_ was lowest (‘best’) given the high-dose CI_6000mg_+LD_1000mg_ regimen in critically ill (sepsis I and II [22,23]) and obese patients [19] (B_tot_P0.95_24h_ = 7.1, 6.6, 7.1 log10 CFU/mL). However, in patients exhibiting lowest concentration-time profiles (neurosurgery patients [18], obese patients [19], patients with CLCR = 250 mL/min [21]), and–when considering the P_0.95_ profile in the population–also in burns patients [20] and a mixed infected population [21], the 3h_TDD6000mg_-infusion regimen was slightly superior, i.e., resulted in lowest bacterial counts after 24 h (although even then bacterial regrowth was observed in the general mixed population [21], in burns patients [20] and neurosurgery patients [18]: B_tot_P0.95_24h_ = 8.3, 7.8, 8.9 log10 CFU/mL).

##### Least Favourable Regimens

*Standard short (0.5-h) infusions* appeared as the least favourable dosing regimen for the ARU552 strain (MIC = 16 mg/L) at 24 h (see Figure S1: standard 0.5h_TDD3000mg_ dosing for the default scenario and Figure S2: 0.5h_TDD6000mg_ versus prolonged and continuous infusion regimens). Continuous infusion without loading dose performed worst with respect to bacterial reduction at 8 h after start of treatment (e.g., in neurosurgery patients), particularly given a low total daily dose (CI_3000mg_) and high-dose scenarios associated with low exposure (e.g., obese patients).

##### Evaluation at 24 h versus 8 h and Impact of a Loading Dose

Addition of a *loading dose* to CI regimens foremost improved bacterial killing at 8 h after start of dosing. CI+LD_1000mg_ resulted in higher bacterial reduction after 8 h than a 3-h_2000mg_ infusion. However, CI+LD_500mg_ notably resulted in lower bacterial reduction after 8 h than a 3-h_2000mg_ infusion for low-exposure scenarios (e.g., CLCR = 250 mL/min) and for P_0.95_ scenarios, despite the higher dose administered during 8 h (2375 mg versus 2000 mg). At 8 h, continuous infusion without loading dose performed worst (together with 0.5-h infusions for some median profiles). B_tot_P0.95_ patterns differed between 8 h and 24 h, insofar as bacterial loads commonly appeared lower at 8 h and differences between the ten studied dosage regimens for the same scenario were often less pronounced at 8 h (see Figure 1 and Figure S3). B_tot_median_ patterns also revealed overall lower bacterial load after 8 h versus 24 h. Contrary to B_tot_P0.95_, higher B_tot_median_ values were obtained for CI + LD_500mg_ when based on the first 8 h versus 24 h (B_tot_median_8h_ > B_tot_median_24h_, but B_tot_P0.95_8h_ < B_tot_P0.95_24h_).

##### Evaluation of Median versus P_0.95_ Profiles 

The results for B_tot_median_24h_ and B_tot_P0.95_24h_ of the 1000 simulated PKPD profiles given MIC = 16 mg/L were similar regarding the dosage regimen producing the highest B_tot_24h_ (0.5-h infusion), whereas for B_tot_P0.95_24h_, a tendency from CI + LD towards 3-h infusions to produce the lowest B_tot_24h_ could be observed.

##### Site of Infection

For scenarios with extensive regrowth (e.g., low-dose regimens in critically ill patients or peripheral sites of infection), no marked difference was observable between the different dosing strategies. For example, the bacterial load after 24 h was high at peripheral infection sites (i.e., lung, cerebrospinal fluid, subcutaneous tissue and peritoneal fluid) and barely allowed for a comparison of dosage regimens; just a slight trend favouring the CI + LD_1000mg_ or the 3-h infusion regimens could be observed.

#### 2.1.2. Pathogen-Related Characteristics

Given the ARU552 strain with MIC = 16 mg/L, continuous infusion with a loading dose (CI + LD_1000mg_) appeared as the most favourable regimen for the median profile of the default scenario (general mixed population [21]). To achieve an as low as possible bacterial load for the P_0.95_ profile in the population, a 3-h infusion regimen seemed more appropriate after 24 h. When assuming lower MIC values and thus higher susceptibility, the same trend (with CI+LD performing well) could be observed (MIC = 8 mg/L: CI_6000mg_; MIC = 4 mg/L: CI_3000mg_). Again, for the EC_50_ = 4.4 mg/L scenario and when looking at the P_0.95_ profile of the population, 3-h infusion regimens became comparable to CI + LD regimens. Interestingly, for pathogens with assumed MIC = 4 mg/L, low-dose 3-h infusions appeared more favourable than high-dose 0.5-h infusions to suppress bacterial growth for the P_0.95_ profile in the population at 24 h. However, 0.5-h infusions performed slightly better than 3-h infusions for high-dose scenarios with EC_50_ = 4.4 mg/L, although for both of these scenarios marked bacterial suppression occurred (B_tot_ for 0.5-h/3-h infusions: 4.7/5.0 log10 CFU/mL). 

At 8 h and for more susceptible strains (MIC = 4–8 mg/L), all CI_6000mg_ regimens–and given MIC = 4 mg/L even CI_3000mg_ + LD_1000mg_ and CI_3000mg_ + LD_500mg_ regimens–were superior to intermittent 0.5-h and 3-h dosing regimens. Consistent with B_tot_P0.95_24h_ values, highest (least favourable) B_tot_median_24h_ values were attained by low-dose continuous-infusion regimens across the diverse scenarios for resistant *P. aeruginosa* with MIC = 16 mg/L, while for pathogens with higher antibacterial sensitivity (MIC = 8 mg/L), continuous-infusion regimens were superior or comparable to intermittent dosing regimens with respect to B_tot_median_24h_.

Modification of the strain virulence, i.e., assumption of a 40% reduction of the growth rate (±death rate) constant resulted in overall lower bacterial counts compared to the default scenario for all dosing regimens. Continuous infusions without loading dose performed worst at 24 h, revealing the importance of high concentrations to suppress bacterial growth for these scenarios. Intermittent 3-h infusions appeared more favourable than continuous infusion after 24 h for the P_0.95_ profile in the population. For the low-dose scenario, 0.5-h infusions resulted in lower bacterial counts than 3-h infusions (B_tot_ for 0.5-h/3-h infusions: 7.6/8.0 log10 CFU/mL). 

When assuming a higher initial bacterial load of 8 log10 CFU/mL instead of 6 log10 CFU/mL, CI_6000mg_ + LD_1000mg_ best suppressed bacterial growth given the median CFU profile in the population, and 3h_TDD6000mg_ performed comparably well after 24 h given the P_0.95_ profile in the population. The same pattern was obtained when assuming a fraction of cells in the resting state at the start of treatment.

In contrast to the resistant ARU strain, the ATCC strain (MIC = 1 mg/L) was markedly suppressed by all ten investigated dosing regimens, with the low-dose standard 0.5-h infusion scheme again resulting in the overall highest bacterial counts (3.6 log10 CFU/mL) for the P_0.95_ CFU profile after 24 h. Assuming higher MIC values (MIC = 4 mg/L and MIC = 8 mg/L) by scaling the slope parameter accordingly suggested that no bacteriostasis was achieved after 24 h with any investigated dosing regimen. For MIC = 2 mg/L and MIC = 4 mg/L, low-dose standard 0.5-h infusions performed worst with respect to bacterial killing after 24 h, whereas high-dose CI_6000mg_ + LD_1000mg_ infusions performed best. Similarly, CI_6000mg_ + LD_1000mg_ performed best and standard 0.5-h_3000mg_ infusions worst for all MIC = 1–8 mg/L at 8 h (rather than 24 h) after start of treatment.

### 2.2. Evaluation of fT_>MIC_

Apart from bacterial load, the ten different dosing regimens were additionally evaluated with respect to *f*T_>MIC_ values, thus merely considering the shape of the concentration-time profile and the minimum inhibitory concentration of the pathogen. 

#### 2.2.1. Patient-Related Characteristics and Site of Infection 

Given MIC = 16 mg/L of the resistant strain, all five low-dose dosing regimens resulted in insufficient *f*T*_>MIC_* values (<20%) at 24 h for all median concentration-time profiles of the six investigated patient populations. Only when assuming renal impairment (CLCR = 30 mL/min) or higher bacterial susceptibility (EC_50_ = 4.4 or 8.8 mg/L) were higher *f*T_>MIC_ values (>30%) reached. For these three scenarios, CI_6000mg_ + LD_1000mg_ appeared as the most favourable regimen (resulting in highest *f*T_>MIC_ values), even when considering the P_0.95_ concentration-time profile in the population.

##### Most Favourable Regimens

For most scenarios, the high-dose continuous-infusion regimens CI_6000mg_ + LD_1000mg_ performed best when considering the median pharmacokinetic profile of the patient population (*f*T_>MIC_median_24h_). In the case of two exceptions, i.e., the presence of high clearance (neurosurgery and obese patients) and CLCR = 250 mL/min (mixed adult population), intermittent infusion regimens (3-h > 1-h) performed better. The *f*T_>MIC_ value reached by the P_0.95_ concentration-time profile in the population was highest (at both 24 h and 8 h) given the high-dose 3h_TDD6000mg_ infusion regimen (*f*T_>MIC_PI0.95_24h_ = 38.1% (sepsis II patients [22])–27.6% (neurosurgery patients [18])), followed by the 0.5 h_TDD6000mg_ infusion regimen. Thus, for example, 95% *f*T_>MIC_ values of the simulated critically ill population described by Roberts et al. [22] were ≥38%, whereas the probability to attain a target of *f*T_>MIC_ = 40% appeared largely insufficient (≪90%) in the neurosurgery patient population with meningitis [18].

##### Least Favourable Regimens

Given the typical patient features of the six populations, low-dose CI_3000mg_ regimens (even with loading dose) resulted in the lowest *f*T_>MIC_P0.95_ values of all investigated dosing scenarios. In contrast, high-dose CI_6000mg_ regimens resulted in the highest *f*T_>MIC_P0.95_ values, if infected patients displayed impaired renal function (CLCR = 30 mL/min, mixed adult population).

##### Evaluation at 24 h versus 8 h and Impact of a Loading Dose

*f*T_>MIC_P0.95_ and *f*T_>MIC_median_ patterns did not differ considerably when based on 24 h or 8 h (Figure 1 and Figure S3). Continuous-infusion regimens with loading dose resulted in slightly higher *f*T_>MIC_P0.95_ when based on the first 8 h versus 24 h. Contrary to *f*T_>MIC_P0.95_, CI+LD_500mg_ resulted in lower *f*T_>MIC_median_ when based on the first 8 h versus 24 h.

##### Evaluation of Median versus P_0.95_ Profiles

In summary, results for the median and P_0.95_ of the 1000 simulated PKPD profiles were similar regarding the dosage regimen producing the lowest relative *f*T_>MIC_ (low-dose continuous infusion), but differed markedly regarding the highest relative *f*T_>MIC_ given MIC = 16 mg/L (*f*T_>MIC_median_24h_: high-dose CI (except neurosurgery and obese patients), *f*T_>MIC_P0.95_24h_: 3-h infusion).

#### 2.2.2. Pathogen-Related Characteristics

Of all pathogen-related characteristics investigated, solely the minimum inhibitory concentration affects *f*T_>MIC_. High-dose continuous-infusion regimens were associated with higher *f*T_>MIC_P0.95_ and *f*T_>MIC_median_24h_ than intermittent dosing regimens for pathogens with assumed higher sensitivity (EC_50_ = 4.4 mg/L and 8.85 mg/L~MIC = 4 mg/L and 8 mg/L). CI_3000-6000mg_ regimens (MIC = 4 mg/L) or CI_6000mg_ regimens (MIC = 8 mg/L) overall performed best.

The lowest *f*T_>MIC_median_24h_ values were attained by low-dose continuous-infusion regimens (without loading dose) across the diverse scenarios for resistant *P. aeruginosa* with MIC = 16 mg/L. Just for pathogens with higher antibacterial sensitivity (MIC = 4–8 mg/L), all (including low-dose) continuous-infusion regimens were superior to intermittent dosing regimens with respect to *f*T_>MIC_median_24h_ and all high-dose continuous-infusion regimens were superior with respect to *f*T_>MIC_P0.95_24h_.

Previous studies have suggested exceeding the MIC by four times (and using *f*T_>4∙MIC_), particularly for severely ill patients. When targeting *f*T_>4∙MIC_, values of *f*T_>4∙MIC_ < 20% were observed for all scenarios unless assuming higher susceptibility (MIC = 4 mg/L; see Figure S4).

### 2.3. Comparison fT_>MIC_ and Total Bacterial Load (B_tot_) 

*f*T_>MIC_ showed higher consistency between 8 h and 24 h than B_tot_ (Figure 1 and Figure S3). A discrepancy between conclusions based on *f*T_>MIC_ and B_tot_ was more pronounced at 8 h than at 24 h, particularly for dosing regimens including a loading dose. For example, *f*T_>MIC_P0.95_8h_ was highest for 3h_TDD6000mg_ for most scenarios, whereas B_tot_P0.95_8h_ was lowest (‘best’) for the CI_6000mg_ + LD regimen (Figure S3). Administration of a loading dose foremost improved bacterial killing and B_tot_ at 8 h but resulted in similar *f*T_>MIC_ compared to other dosing regimens. 

**Figure 2:**
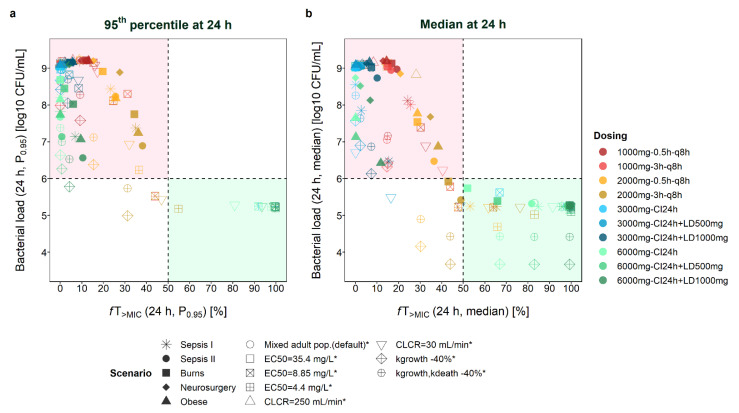
Total bacterial load versus *f*T_>MIC_ (time that meropenem concentrations exceed the minimum inhibitory concentration) reached by 95% (P_0.95_, panel **a**) or 50% (median, panel **b**) of the patient population at 24 h after start of therapy given different scenarios. Sepsis I: Delattre et al. [23]; Sepsis II: Roberts et al. [22] (no renal dysfunction); EC_50_: drug concentration that produces 50% of E_max_ (maximum achievable kill rate constant); CLCR: creatinine clearance; k_growth_: rate constant of bacterial growth; k_death_: rate constant of natural bacterial death; higher inoculum: initial bacterial load of 8 log10 CFU/mL, either assumed to be susceptible or with an initial fraction in the resting state; scenarios marked with an asterisk * refer to a mixed adult population (default scenario; Li et al. [21]); CI: continuous infusion; LD: loading dose; q8h: every eight hours.

For a dosing regimen to be considered adequate, suppression of bacterial growth (i.e., at least bacteriostasis) or, when referring to PK/PD indices, a target of at least *f*T_>MIC_ = 50% has been suggested for clinical settings. Figure 2 compares the attainment of these two thresholds for scenarios with an initial inoculum of 6 log CFU/mL (targets: bacterial load ≤ 6 log10 CFU/mL and *f*T_>MIC_ ≥ 50%). It becomes evident that even when the same *f*T_>MIC_ values were obtained for several scenarios, markedly different B_tot_ values could be observed. When looking at median profiles at 24 h after start of dosing (Figure 2b), all scenarios leading to bacterial growth (>6 log10 CFU/mL) were associated with *f*T_>MIC_ < 50%. In other words, all scenarios leading to *f*T_>MIC_>50% (even *f*T_>MIC_ > 40%) over 24 h resulted in bacteriostasis. For most scenarios, *f*T_>MIC_ values between 20% and 50% resulted in a higher bacterial load after 24 h compared to the start of treatment. However, for some scenarios, bacterial suppression was achieved even for lower *f*T_>MIC_ values, i.e., for a mixed adult population with CLCR = 30 mL/min and CI_3000mg_ + LD_500mg_ dosing (*f*T_>MIC_ = 16.5%, B_tot_ = 5.5 log10 CFU/mL), for scenarios assuming a 40% lower growth rate and high-dose intermittent infusion (3h_TDD6000mg_, 0.5h_TDD6000mg_), and for several scenarios with *f*T_>MIC_ = 40–50% (e.g., adult patient with CLCR = 30 mL/min and CI_3000mg_ + LD_1000mg_ dosing: *f*T_>MIC_ = 48.6%, B_tot_ = 5.3 log10 CFU/mL; median profile for burns/critically ill (sepsis II [22])/mixed adult population and 3h_TDD6000mg_ dosing; scenarios with EC_50_ = 4.4 mg/L and 0.5-h dosing, or EC_50_ = 8.8 mg/L and 3-h dosing).

The assessment of P_0.95_ profiles rather than median profiles revealed a similar picture, with most scenarios resulting in insufficient drug exposure and bacterial killing (Figure 2a). Scenarios leading to *f*T_>MIC_ < 50% but still B_tot_ < 6 log10 CFU/mL again included scenarios assuming a lower growth rate of the pathogen (high-dose dosing regimens with prolonged infusions), and scenarios with EC_50_ = 4.4 mg/L (3h_TDD6000mg_), EC_50_ = 8.8 mg/L (3h_TDD6000mg_), and CLCR = 30 mL/min (3h_TDD6000mg_).

At 8 h after the start of treatment (Figure S5), more scenarios led to B_tot_ < 6 log10 CFU/mL but *f*T_>MIC_ < 50%, including scenarios with lower growth rates and beyond (e.g., burns patients: CI_6000mg_+LD_500mg_ at 8 h: *f*T_>MIC_ = 19.6%/B_tot_ = 5.7 log10 CFU/mL, at 24 h: *f*T_>MIC_ = 65.9%/B_tot_ = 5.4 log10 CFU/mL). Last, *f*T_>4∙MIC_ values > 50% were only attained when assuming EC_50_ = 4.4 mg/L (Figure S4). Thus, stasis was also observed for some scenarios with *f*T_>4∙MIC_ < 20% (Figure S6).

## 3. Discussion

Our study applied PKPD models to enable in vivo predictions of antibiotic concentrations and bacterial killing (i) for clinically relevant scenarios other than originally studied (‘what-if’ scenarios, e.g., in specific groups of patients, given specific patient characteristics, in specific compartments of the body or given novel dosage regimens); (ii) at any time point of interest; and (iii) assuming different bacterial susceptibility. These simulations can aid in identifying and pre-selecting promising scenarios to be investigated in clinical studies (e.g., resource-intensive randomised controlled trials). Furthermore, once a dosing scheme has been shown to be efficient in a group of patients, PKPD models could help to identify dosing regimens leading to a similar bacterial pharmacodynamic profile in a different target population. 

Prolonged and continuous infusions have increasingly been promoted and used for antibiotics with ‘time-dependent’ activity, although its impact on clinical outcome is still a subject of research. A meta-analysis of 22 randomised trials associated prolonged infusions (≥3 h) of antipseudomonal beta-lactams with lower all-cause mortality than short-term infusion (≤60 min) for patients with sepsis [24]. Similarly, two smaller meta-analyses reported a significant reduction of hospital mortality or improvement of clinical cure when using >1-h infusions in studies published in or after 2015 [25] and lower mortality for extended infusions (≥3 h) [26]. However, most of these studies did not differentiate between different types of prolonged infusions (e.g., 3-h or continuous infusion). Simulations with PKPD models allow for the systematic investigation of what-if scenarios, e.g., various lengths of infusions with or without a loading dose, and can help to create hypotheses regarding potentially efficacious dosing regimens. For example, the present analysis compared five different modes of administration, both as high-dose and low-dose regimens administered as 0.5-h, 3-h and continuous-infusion regimens (with and without a loading dose).

In our study, conclusions regarding which of these ten investigated intermittent and prolonged-infusion regimens seemed most favourable for a resistant *P. aeruginosa* strain (MIC = 16 mg/L) depended on several factors: First, the surrogate of efficacy chosen as the basis for comparison of dosing regimens played a role, i.e., total bacterial load (B_tot_) as a result of meropenem exposure (using the PKPD model), or *f*T_>MIC_ (based on a PK/PD index-based approach, using merely the PK component of the model and MIC information), which was evaluated in the context of previously determined PK/PD targets as thresholds for efficacy. For instance, the benefit of a loading dose after 8 h became more apparent in B_tot_ than *f*T_>MIC_. Given the P_0.95_ profile in the population, CI + LD_1000mg_ appeared most favourable with respect to bacterial load at 8 h and 24 h for most scenarios, though 3h_TDD6000mg_ performed best with respect to *f*T_>MIC_. The least favourable dosing regimens also differed depending on the measure of evaluation (B_tot_P0.95_24h_: 0.5-h infusion versus *f*T_>MIC_P0.95_24h_: CI without loading dose).

Second, the time of assessing B_tot_ or *f*T_>MIC_ was crucial. For example, an initial effect of a loading dose on the bacterial load at 8 h was outweighed by regrowth at 24 h after the start of antibiotic treatment for some scenarios. Early antibiotic treatment is crucial to minimize the initial bacterial burden [27]. The urgency of early effective antibiotic treatment depends on the clinical condition of the patient and the acuteness of the bacterial infection [28,29].

Third, the most appropriate dosing regimen for a scenario differed depending on whether the PKPD profiles representing the median or the 95th percentile of the population were studied. This observation is important when mimicking human exposure in in vitro time-kill experiments (e.g., hollow-fibre studies). These are often based on the concentration-time profile of the typical patient, i.e., the median representative in a population that was previously determined by a population pharmacokinetic model. The results of the present study call to mind to also consider variability in the population and, for example, to investigate PK(/PD) profiles representing the 95th percentile of a population.

In our study, high total daily dose and shorter infusion durations (3-h_6000mg_) tended to become advantageous for scenarios associated with lower meropenem concentrations (CLCR = 250 mL/min, high clearance) and consequently when assessing the 95th percentile of the pharmacokinetic and pharmacodynamic profiles (B_tot_, *f*T_>MIC_), lower bacterial susceptibility (MIC ≥ 16 mg/L) and assumed lower growth (±death) rate. Hence, the lower the antibiotic concentrations in relation to the MIC were, the more important higher doses or, in case of unchanged doses, the height of concentration peaks following short infusions became. In contrast, continuous infusion appeared to become more favourable for scenarios associated with higher meropenem concentrations (CLCR = 30 mL/min), assessment of the median *f*T_>MIC_ and B_tot_, and higher bacterial susceptibility (MIC ≤ 8 mg/L). Notably, prolonged-infusion dosing like 3-h and CI + LD regimens were largely superior to the approved standard intermittent 0.5-h infusions administered every eight hours.

Our study investigated total daily doses of 3000 mg, corresponding to the approved standard dosing regimen for infections caused by *P. aeruginosa* (1000 mg every eight hours) [3], up to clinically feasible total daily doses of 6875 mg (CI_6000mg_ + LD_1000mg_). The higher total daily dose on the first day compared to the following days of antibiotic treatment (e.g., 6875 mg versus 6000 mg) was owing to assumed unchanged infusion rates after the loading dose across the entire treatment period, as clinically feasible and previously accepted in randomised controlled trials [30]. Maximum doses of 6000 mg/day, e.g., three times 2000 mg over 0.5 h or 3 h for meningitis, have previously been recommended [11]. Apart from that, off-label continuous regimens with 6000 mg daily preceded by loading doses of 2000 mg, or even 3500 mg every 6 h, have been reported [31,32]. 

In our simulation study, median concentrations up to 30.9 mg/L (CLCR = 30 mL/min; CI_6000mg_), followed by 24.6 mg/L (critically ill, Sepsis I [23]; CI_6000mg_) were simulated at 24 h and the corresponding concentrations covering 90% of the population were 18.2–55.0 mg/L and 24.6–47.3 mg/L, respectively. An arbitrary safety threshold of 100–120 mg/L, thus higher than the meropenem concentrations simulated in our study, has previously been suggested to be tolerable [33]. A retrospective study on beta-lactam toxicity during intermittent infusions associated minimum concentrations > 64.2 mg/L and >44.5 mg/L with a 50% risk of developing neurotoxicity and nephrotoxicity, respectively [34]. Another retrospective review of critically ill patients receiving meropenem doses of ≤6000 mg/day did not identify additional toxicities [35].

Varied pathogen susceptibility (different MIC values), also reflecting potential uncertainty in the MIC determination was mimicked by scaling EC_50_ accordingly. The rationale for this approach, i.e., the ability of the model to predict new strains based on their MIC only, has previously been shown for both other mutants and clinical isolates [36]. The MIC was assumed to be constant over the investigated 24-h observation period. For longer clinical trials, regular MIC measurements (rather than baseline only) could be valuable.

Lower growth and death rates have been demonstrated in vivo compared to in vitro [6], though these differences also depend on the mechanism of action. Given a 40% reduced growth (±death rate) for the resistant *P. aeruginosa* strain, the same antibiotic dose appeared more effective compared to the default strain. Further studies are warranted to confirm this finding specifically for meropenem and strains with slower bacterial growth. 

Most scenarios of our analysis considered plasma concentration-time profiles and thus strictly bloodstream infections. For four populations (critically ill, neurosurgery and obese patients), peripheral locations of infection (lung, cerebrospinal fluid, interstitial space fluid, peritoneal fluid) were additionally investigated. However, the comparison of dosing regimens for these peripheral sites of infection was not straightforward due to a predicted overall high bacterial load, and no difference could be identified between models capturing both rate and extent, and those capturing only the extent of antibiotic exposure.

Some limitations of our analysis shall be acknowledged. The focus of the present work lies in comparisons of total bacterial load following different dosage regimens relative to a reference dosage regimen and relative to a reference population/scenario–rather than based on absolute bacterial counts (e.g., whether bacteriostasis is reached or not). Details of a bacterial infection like bacterial load at the start of treatment or bacterial growth and death rates might not be directly transferable from in vitro to in vivo scenarios, e.g., due to a different supply of nutrients. Bacterial density in humans might be variable depending on the type of infection or disease severity. Our analysis assumed an initial bacterial load of 6 log10 CFU/mL in agreement with in vitro experiments, or an inoculum of 8 log10 CFU/mL at the start of meropenem treatment [36]. Previous studies suggest that this magnitude might also be plausible for humans (2–10 log10 CFU/mL), as demonstrated in patients with suspected ventilator-associated pneumonia (6.2 log10 CFU/mL in bronchoalveolar lavage fluid [37]), diabetic foot ulcers [38] and patients receiving platelet transfusions [39].

The reliability of the transferability of in vitro growth characteristics to human in vivo conditions to achieve a direct translation of the bacterial burden in patients is still a subject of research. One study, for example, analysed the same bacterial strain in vitro and in vivo and showed that model parameters may need to be adjusted to enable quantitative translation of the bacterial concentrations [40]. However, PKPD models allow for the drawing of inferences regarding which dosing regimen and shape of human concentration-time profile influence bacterial killing in the most favourable way. To verify efficacious and safe dosing in challenging clinical situations with special and highly variable patient populations, poorly susceptible pathogens and long treatment durations, model-informed precision dosing has been proven promising in several studies [41].

Selected scenarios were investigated based on one PKPD model. Different PKPD models might result in different conclusions and further conceivable scenarios could be interesting to investigate, e.g., antibiotic combinations. As the pharmacodynamic component of the PKPD model relied on in vitro time-kill curves, potential effects of the immune system on the bacterial dynamics were not considered in the present simulations. In this respect, the simulated scenarios might represent ‘worst-case’ scenarios in terms of bacterial eradication. Consistent with the in vitro experiments underlying the PKPD model for meropenem and to avoid extrapolation, our simulation study merely covered two strains of *P. aeruginosa* and the first day of treatment, not least due to the importance of early adequate therapy, particularly in patients with severe infections [29,42]. In scenarios with *f*T_>MIC_ values of close to 100%, no regrowth was observed in the first 24 h; however, regrowth could not be excluded for longer durations of observation. As emergence of resistance might occur after 24 h, conclusions might change when evaluating longer periods of antibiotic treatment, e.g., one week of therapy. Hollow-fibre studies enable the observing of time-kill patterns for longer periods of time and could provide the basis for such extended PKPD studies [43].

## 4. Materials and Methods

The objectives were exemplified by a PKPD model describing meropenem effects over time against a resistant *P. aeruginosa* strain (ARU552, MIC = 16 mg/L) and a more susceptible *P. aeruginosa* strain (ATCC27853, MIC = 1 mg/L) [42]. The database originated from 24-h static and dynamic in vitro time-kill curve experiments. The PKPD model comprised two bacterial subpopulations, each with compartments for growing and resting bacteria, and included different drug effect models for the two strains (ARU552: sigmoid E_max_ model; ATCC27853: power model). The model has previously successfully been shown to predict data from PK/PD index studies in mice without re-estimation of model parameters [9,44].

Bacterial growth and killing during meropenem exposure were translated to humans, with the drug effect in the model driven by the pharmacokinetic profiles of different patients based on six previously published population pharmacokinetic models (see Table S1). The investigated patient populations comprised adults with intra-abdominal or lung infections [21], critically ill [22,23], burns [20], obese [19], and neurosurgery patients [18], thus reflecting diverse indications of meropenem. Two critically ill populations were included in the analysis, sepsis patients (Clearance CL = 9.87 L/h given creatinine clearance CLCR = 100 mL/min), and sepsis patients without renal dysfunction (CL = 13.6 L/h, median CLCR~100 mL/min) exhibiting overall lower concentrations. Only two-compartment models that had been developed based on a solid data base, i.e., rich in number of patients and/or samples per patient, were selected. Antibiotic concentration-time profiles and bacterial counts over time were simulated based on unbound meropenem concentrations (assuming an unbound fraction of 98% [3]) over 24 h since the start of antibiotic treatment.

A mixed adult infected population (CLCR = 83 mL/min [21]), original pharmacodynamic parameters of the model [42], and an initial bacterial load of 10^6^ colony forming units (CFU) per mL were chosen as a default scenario, which was compared to diverse alternative scenarios. These comprised varied patient-related characteristics (e.g., disease, CLCR values of the default population varied to 30 mL/min and 250 mL/min instead of 83 mL/min). Most scenarios considered plasma concentration-time profiles and thus strictly bloodstream infections. Apart from that, different peripheral sites of infection were investigated, including lung, cerebrospinal or peritoneal fluid and subcutaneous tissue (see Table S1). If covered by the model, differences in both rate and extent of drug distribution compared to the plasma compartment were considered. For example, the model for neurosurgery patients included a separate compartment for cerebrospinal fluid [18]. In the model for obese patients, rapid equilibrium between the central compartment and the compartments for subcutaneous (SC) tissue and peritoneal fluid (PF) was implemented and concentrations of the central compartment (C_CC_) were thus scaled accordingly (C_SC_ = C_CC_∙0.72 and C_PF_ = C_CC_∙0.94). Penetration from plasma to lung was assumed to be 30% in critically ill patients [22,23], as previously reported for critically ill patients with nosocomial [45] and ventilator-associated pneumonia [46].

Apart from patient characteristics, tweaked pathogen-related features of the resistant strain, including EC_50_, growth rate, kill rate, and start inoculum, were investigated. Different MIC values, scaled by varying the EC_50_ value for the resistant *P. aeruginosa* strain or the slope parameter for the ATCC27853 strain accordingly, were simulated with the PKPD model using the pharmacokinetic component of the default (mixed adult) patient population. In line with the in vitro experiments underlying the model, initial bacterial density was set to 6 log10 CFU/mL for most scenarios and bacteria were assumed to be in the susceptible and growing state at the start of treatment. Additional simulations were performed based on a higher initial inoculum of 8 log10 CFU/mL, with all bacteria assumed as susceptible or else with a certain fraction of bacteria in the resting state at the start of treatment. For the latter scenario, 3.4% of bacteria were initially assumed as resting (as this proportion was found in the resting state after an initial bacterial load of 100 CFU/mL had reached 8 log10 CFU/mL after 21.8 h). The growth rate and the death rate constants of the resistant strain were kept unchanged or were lowered by 40%, as had been observed previously in vivo compared to in vitro [6] (see Table S1 for an overview of investigated scenarios).

**Figure 3:**
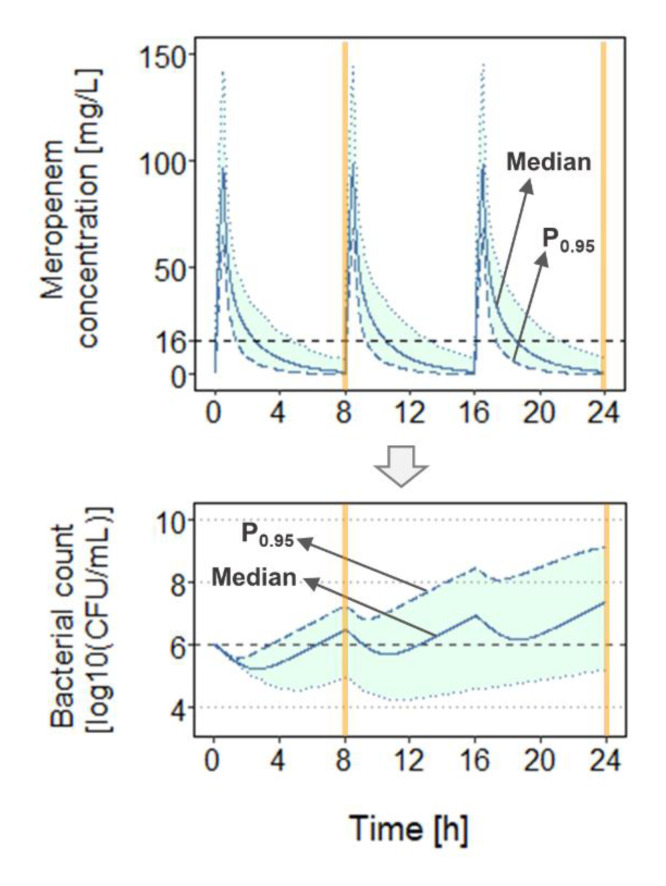
PKPD model-based output assessed for each scenario, exemplified by the default adult infected population (Li et al. [21]) and a 0.5h_TDD6000mg_ (2000 mg every 8 h) dosing regimen. Total bacterial load (B_tot_) and *f*T_>MIC_ (time that unbound meropenem concentrations exceed the minimum inhibitory concentration; here: MIC = 16 mg/L) were assessed at 8 h and at 24 h after start of antibiotic treatment. P_0.95_: profile representing the 95th percentile in the population at a given time point; dashed lines mark the minimum inhibitory concentration of the ARU552 strain (**upper** panel) and the initial bacterial load/bacteriostasis (**lower** panel).

For each scenario, total bacterial load (B_tot_) and *f*T_>MIC_ were determined at 8 h and 24 h after start of dosing (Figure 3) for ten dosage regimens, i.e., total daily doses (TDD) of 3000 mg and 6000 mg, each administered as 0.5-h (0.5h_TDD3000mg_ = 0.5h_1000mg_ every 8 h (q8h), or 0.5h_TDD6000mg_ = 0.5h_2000mg_ q8h), as 3-h (3h_TDD3000mg_ or 3h_TDD6000mg_), or continuous infusion over 24 h without (CI_3000mg_, CI_6000mg_) or with a loading dose (500 mg or 1000 mg as 0.5-h infusion: CI_3000mg_ + LD_500mg_, CI_3000mg_ + LD_1000mg_, CI_6000mg_ + LD_500mg_, CI_6000mg_ + LD_1000mg_). In this work, dosage regimens involving a TDD of 6000 mg (or higher in case of a loading dose) will be referred to as ‘high-dose’ regimens and schemes involving a TDD of 3000 mg as ‘low-dose’ regimens. Continuous-infusion regimens including a loading dose were simulated as usually handled in clinical practice, i.e., with continuous infusion starting directly after the first short infusion (loading dose) with a constant target rate. The present study adopted *f*T_>MIC_ > 50% as a clinical target for efficacy [13] and bacteriostasis as a criterion for evaluating bacterial growth and kill. Simulations (*n* = 1000 for each scenario) were conducted considering between-patient pharmacokinetic variability and using R3.6.1 (mrgsolve package) [47]. For each abovementioned scenario, the median and the 95th percentile (P_0.95_) of the concentration-time profiles (and *f*T_>MIC_ values), as well as of the bacterial count-time profiles (and B_tot_ values) were investigated (Figure 3). P_0.95_ was chosen not least as the 95th percentile of a study population is commonly used in simulations underlying the probability of target attainment (PTA) analyses to determine efficacious antibiotic doses.

## 5. Conclusions

We present a showcase platform exemplified by meropenem and *P. aeruginosa* to illustrate how semi-mechanistic PKPD models can serve to translate in vitro antibiotic efficacy over time to different real-world clinical situations. PKPD models for antibiotic effects may be used to investigate a wide range of dosing scenarios (which would require an infeasibly high number of clinical trials) and help to identify the most promising strategies for efficacious dosing, also taking into account factors beyond pharmacokinetics. Our analysis identified continuous-infusion regimens preceded by a 1000 mg-loading dose as well as 3-h intermittent infusions q8h as superior to standard 0.5-h infusions q8h 24 h after start of therapy. The optimal infusion duration might vary depending on the individual patient to be treated and decision support tools may facilitate its selection in the future. The present analysis serves as a reminder that not only the total daily dose but also the infusion duration deserves special attention in dosing individualisation.

## Data Availability

The present manuscript presents a simulation study based on previously published population pharmacokinetic and pharmacodynamic models and did not involve any data collection.

## Supplementary Materials

The following supporting information can be downloaded at: https://www.mdpi.com/article/10.3390/antibiotics11081036/s1, Table S1. Overview of the population pharmacokinetic models used to inform the pharmacokinetic/pharmacodynamic (PKPD) model; Table S2. Total bacterial load at 8 h and 24 h after start of therapy based on median PKPD profiles; Table S3. *f*T_>MIC_ at 8 h and 24 h after start of therapy based on median PKPD profiles; Figure S1. Concentration-time profile of meropenem and resulting bacterial load over time for a resistant *Pseudomonas aeruginosa* strain given approved standard dosing of meropenem (1000 mg every 3 h administered as 0.5-h infusions); Figure S2. Concentration-time profiles of meropenem and resulting bacterial load over time for a resistant *Pseudomonas aeruginosa* strain in six different patient populations given three dosing regimens: (i) 2000 mg every 8 h administered as 0.5-h infusions, (ii) 2000 mg every 8 h administered as 3-h infusions, and (iii) 6000 mg/24 h administered as continuous infusion following a loading dose of 1000 mg; Figure S3: Total bacterial load and *f*T_>MIC_ (time that meropenem concentrations exceed the minimum inhibitory concentration) reached by 95% or 50% of the patient population at 8 h after start of therapy given different scenarios; Figure S4. *f*T_>4∙MIC_ (time that meropenem concentrations exceed four times the minimum inhibitory concentration) reached by 95% or 50% of the patient population at 24 h after start of therapy given different scenarios; Figure S5: Total bacterial load versus *f*T_>MIC_ (time that meropenem concentrations exceed the minimum inhibitory concentration) reached by 95% or 50% of the patient population at 8 h after start of therapy given different scenarios; Figure S6: Total bacterial load versus *f*T_>4∙MIC_ (time that meropenem concentrations exceed four times the minimum inhibitory concentration) reached by 95% or 50% of the patient population at 24 h and 8 h after start of therapy given different scenarios.
