# Supplementary material for: Model-Informed Translation of In Vitro Effects of Short-, Prolonged- and Continuous-Infusion Meropenem against Pseudomonas aeruginosa to Clinical Settings
Source: Antibiotics (Basel). 2022 Aug 1;11(8):1036. doi: 10.3390/antibiotics11081036 (PMC9404958; doi:10.3390/antibiotics11081036)
Supplement: Supplementary file 1 [file antibiotics-11-01036-s001.zip › antibiotics-1799773-supplementary.pdf]

antibiotics

Supplementary data

Model-informed translation of *in vitro* effects of short-, prolonged- and continuous-infusion meropenem against *Pseudomonas aeruginosa* to clinical settings

Iris K. Minichmayr, Suzanne Kappetein, Margreke J.E. Brill and Lena E. Friberg *

Department of Pharmacy, Uppsala University, Box 580, 75123 Uppsala, Sweden
* Correspondence: lena.friberg@farmaci.uu.se; Tel.: +46 18 471 4685

Table S1. Overview of the population pharmacokinetic models used to inform the pharmacokinetic/pharmacodynamic (PKPD) model (bold scenarios mark the default settings adopted for simulations of other populations).

Ref.	Population	Covariates, parameters	Dosing	Sampling	Simulated scenarios
Li et al. 2006	Adults with intra-abdominal infections, CAP or VAP (n=79)	$CLCR_{IBW}$_CL, Age_CL, WT_V (CLCR=83 mL/min, Age=35 y, WT=70 kg) CL=14.6 L/h, V1=10.8 L, V2=12.6 L, Q=18.6 L/h	500/1000/ 2000 mg, 0.5/3 h, q8h	3rd dose interval or steady state, n=341	Plasma **k_{growth}, k_{death} 100%**, -40% **Initial bacterial load 10^6** and 10^8 CFU/mL **ARU551 (MIC 16 mg/L):** EC_{50} 4.4-35.4 mg/L ATCC27853 (MIC 1 mg/L) CLCR 30/250 mL/min
Delattre et al. 2012	Patients with severe sepsis or septic shock (n=19)	CLCR_CL (CLCR=100 mL/min) WT_all parameters (WT=70 kg) CL=9.87 L/h, V1=24.4 L, V2=7.01 L, Q=4.97 L/h	1000 mg, 0.5 h, q8h	1, 1.5, 4.5, 6 or 8, 24 h, first 24 h of therapy	Plasma, lung (30% exposure)
Roberts et al. 2009	Critically ill with sepsis, no renal dysfunction (n=10)	CLCR_CL (CLCR=100 mL/min) CL=13.6 L/h, V1=7.9 L, V2=14.8 L, Q=56.3 L/h	1500 mg (1st dose), then 1000 mg q8h; 3000 mg/24 h + LD 500 mg	Day 1: 0-8 h (15x) Days 2-5: 0-8 h (9x) n=222	Plasma, lung (30% exposure)
Doh et al. 2010	Burns patients (n=59)	CLCR_CL, Oedema_V1 (CLCR=135 mL/min) CL=4.45 L/h, V1=17.0 L, V2=10.1 L, Q=5.25 L/h	500-1000 mg, 0.5 h, q8h/q12h	0, 1, 2, 4, 6, 8, 9, 24 and 25 h (after start of 4th dose)	Plasma
Wittau et al. 2010	Morbidly obese, intraperitoneal surgery (n=6)	Fat free mass on all parameters (FFM=53 kg) CL=18.7 L/h, V1=21.5 L, V2=6.16 L, Q=29.4 L/h, FSC=0.721, FPF=0.943	1000 mg, 15 min, q8h	≤8h (0.5, 1, 2, 3, 5, 8 h) after 4th dose	Plasma, subcutaneous tissue (SC), peritoneal fluid (PF)
Lu et al. 2015	Patients with meningitis after neurosurgery (n=82)	No covariates CL=22.2 L/h, V1=17.9 L, V2=3.84 L, Q=1.79 L/h, VCSF=0.13 L, QCSF=0.01 L/h, PC=0.172	1000 mg, q8h/q6h, 2000 mg, 1 g/h, q8h	during inf., 0.17, 2, 4 h after, or: at inf. end, 1 h, 3 h after end, before next	Plasma, cerebrospinal fluid (CSF)

Abbreviations: Ref.: Reference, CAP: community-acquired pneumonia; VAP: ventilator-associated pneumonia; CLCR: creatinine clearance; $CLCR_{IBW}$: CLCR calculated based on ideal body weight; WT: total body weight; CL: clearance; V1/V2: central/peripheral volume of distribution; Q: intercompartmental clearance; FSC/FPF: factors quantifying penetration of meropenem in subcutaneous tissue and peritoneal fluid; CSF: cerebrospinal fluid; PC: transfer multiplier between central and CSF compartment; q6h/q8h/q12h: every 6/8/12 h; LD: loading dose; inf.: infusion; k_{growth}/k_{death}: growth/death rate constant in PKPD model; CFU: colony-forming unit.

Table S2. Total bacterial load at 8 h and 24 h after start of therapy based on median PKPD profiles.

				**Total bacterial load (B_{tot}, median profile)**									
				**TDD ~3000 mg/day**					**TDD ~6000 mg/day**				
				**II 1000 mg q8h**		**CI 3000 mg/24h**			**II 2000 mg q8h**		**CI 6000 mg/24h**		
**Model**	**Population**	**Scenario**	**B_{tot} time**	**0.5h**	**3h**	**no LD**	**LD 500**	**LD 1000**	**0.5h**	**3h**	**no LD**	**LD 500**	**LD 1000**
Li 2006	Infected adults	Default (Plasma, CLCR 83 mL/min, MIC 16 mg/L)	8 h	7.29	7.28	7.79	7.13	6.40	6.49	**5.95**	6.30	**5.59**	**4.92**
			24 h	9.14	9.04	9.02	9.07	9.00	7.39	**5.78**	**5.62**	**5.34**	**5.27**
Doh 2010	Burns	Plasma	8 h	7.27	7.31	7.82	7.19	6.46	6.56	6.03	6.40	**5.69**	**5.03**
			24 h	9.13	9.05	9.02	9.07	9.03	7.54	**5.92**	**5.74**	**5.39**	**5.28**
Delattre 2012	Sepsis	Plasma	8 h	6.90	6.97	7.58	6.62	**5.45**	**5.44**	**5.21**	**5.82**	**4.99**	**4.26**
			24 h	8.12	8.00	8.55	7.85	6.47	**5.25**	**5.24**	**5.24**	**5.24**	**5.24**
		Lung	8 h	8.06	8.12	8.16	8.11	7.95	7.61	7.77	8.02	7.93	7.70
			24 h	9.05	9.04	9.04	9.05	9.06	9.08	9.02	9.01	9.02	9.05
Roberts 2009	Sepsis	Plasma	8 h	7.12	7.18	7.72	6.98	6.09	6.18	**5.73**	6.11	**5.33**	**4.64**
			24 h	8.97	8.94	9.00	9.02	8.73	6.47	**5.42**	**5.32**	**5.26**	**5.25**
		Lung	8 h	8.06	8.13	8.17	8.13	7.99	7.66	7.85	8.06	8.00	7.82
			24 h	9.05	9.05	9.05	9.05	9.06	9.16	9.04	9.04	9.05	9.06
Lu 2016	Neurosurgery	Plasma	8 h	7.51	7.65	8.02	7.65	7.15	7.03	6.58	7.22	6.77	6.25
			24 h	9.20	9.07	9.04	9.08	9.13	8.85	7.68	8.74	8.52	8.13
		Cerebrospinal fluid	8 h	8.18	8.18	8.18	8.18	8.18	8.18	8.18	8.18	8.18	8.18
			24 h	9.05	9.05	9.05	9.05	9.05	9.05	9.05	9.05	9.05	9.05
Wittau 2015	Obese	Plasma	8 h	7.39	7.56	7.95	7.51	6.81	6.60	6.32	6.91	6.36	**5.63**
			24 h	9.19	9.07	7.04	9.09	9.16	7.77	6.87	7.65	7.13	6.42
		s.c. interstitial space fluid	8 h	7.68	7.87	8.08	7.87	7.37	6.97	6.92	7.55	7.24	6.66
			24 h	9.15	9.04	9.04	9.06	9.12	8.72	8.56	8.97	8.97	8.83
		Peritoneal fluid	8 h	7.43	7.60	7.97	7.56	6.89	6.65	6.39	7.01	6.49	**5.77**
			24 h	8.42	8.35	8.71	8.53	8.39	7.76	6.96	7.21	6.92	6.55
Li 2006	Infected adults	CLCR 30 mL/min	8 h	6.44	6.33	7.03	**5.61**	**4.53**	**4.96**	**4.54**	**4.89**	**4.17**	**3.95**
			24 h	6.89	6.23	6.71	**5.49**	**5.26**	**5.22**	**5.23**	**5.24**	**5.24**	**5.24**
		CLCR 250 mL/min	8 h	7.72	7.89	8.10	7.87	7.53	7.36	7.03	7.68	7.38	6.99
			24 h	9.15	9.05	9.05	9.07	9.10	9.18	8.82	9.03	9.05	9.07
		EC_{50} 4.4 mg/L	8 h	**5.50**	**4.71**	**4.19**	**3.90**	**3.85**	**4.51**	**3.98**	**3.88**	**3.85**	**3.80**
			24 h	**5.72**	**5.22**	**5.24**	**5.23**	**5.19**	**4.69**	**5.02**	**5.16**	**5.15**	**5.10**
		EC_{50} 8.8 mg/L	8 h	6.49	**5.95**	6.30	**4.92**	**4.34**	**5.51**	**4.71**	**4.20**	**3.95**	**3.90**
			24 h	7.39	**5.78**	**5.62**	**5.27**	**5.25**	**5.23**	**5.22**	**5.24**	**5.24**	**5.23**
		EC_{50} 35.4 mg/L	8 h	7.82	8.00	8.12	7.97	7.62	7.29	7.28	7.79	7.57	7.13
			24 h	9.11	9.04	9.04	9.06	9.09	9.14	9.04	9.02	9.04	9.07
		k_{growth}, k_{death} -40%	8 h	6.41	6.40	6.92	6.25	**5.52**	**5.61**	**5.07**	**5.43**	**4.73**	**4.14**
			24 h	7.16	7.06	8.26	7.64	6.87	**4.90**	**4.43**	**4.43**	**4.42**	**4.42**
		k_{growth} -40%	8 h	6.16	6.15	6.67	**6.00**	**5.27**	**5.36**	**4.82**	**5.18**	**4.48**	**3.98**
			24 h	6.42	6.32	7.57	6.90	6.13	**4.16**	**3.68**	**3.68**	**3.67**	**3.67**
		Higher initial bact. load (S)	8 h	9.01	8.97	9.11	8.85	8.31	8.41	7.90	8.19	7.57	6.95
			24 h	8.97	8.89	8.92	8.92	8.97	8.75	7.63	7.26	7.19	7.20
		Higher initial bact. load (SR)	8 h	9.00	8.96	9.10	8.84	8.30	8.40	7.88	8.18	7.56	6.98
			24 h	8.96	8.89	8.92	8.92	8.97	8.75	7.62	7.26	7.19	7.20

Abbreviations: TDD: total daily dose; II: intermittent dosing; CI: continuous infusion; q8/24h: every 8/24 hours; LD: loading dose (in mg); CLCR: creatinine clearance; MIC: minimum inhibitory concentration; s.c. subcutaneous; EC_{50}: drug concentration (mg/L) that produces 50% of maximum achievable kill rate constant; kgrowth: rate constant of bacterial growth; kdeath: rate constant of natural bacterial death; higher initial bacterial load: 8 log10 CFU/mL, either assumed to be susceptible (S) or with an initial fraction in the resting state (SR); categories/colour code (scenarios with initial bacterial load 6 log10 CFU/mL): bacteriostasis/net bacterial kill (bold values): >5-6 log10 CFU/mL (beige), >4-5 log10 CFU/mL (light cyan), ≤4 log10 CFU/mL (mint); net bacterial growth (non-bold values): >6-7 log10 CFU/mL (light red), >7-8 log10 CFU/mL (lilac), >8 log10 CFU/mL (violet red). Colours were intended to be accessible to people who are colour-blind (https://davidmathlogic.com/colorblind; access date 26.07.2022).

Table S3. $fT_{>MIC}$ at 8 h and 24 h after start of therapy based on median PKPD profiles.

Model	Population	Scenario	$fT_{>MIC}$ time	$fT_{>MIC}$ (median profile): TDD ~3000 mg/day: II 1000 mg q8h: 0.5h	3h	CI 3000 mg/24h: no LD	LD 500	LD 1000	TDD ~6000 mg/day: II 2000 mg q8h: 0.5h	3h	CI 6000 mg/24h: no LD	LD 500	LD 1000
Li 2006	Infected adults	Default (Plasma, CLCR 83 mL/min, MIC 16 mg/L)	8 h	14.6	12.2	0	6.7	*21.9*	*29.6*	**42.8**	0.7	**51.8**	**98.1**
			24 h	14.9	14.6	0	2.2	7.3	*30.2*	**43.9**	**66.9**	**82.9**	**99.3**
Doh 2010	Burns	Plasma	8 h	16.5	12.9	0	7.7	*22.3*	*27.9*	**41.7**	0	19.6	**98.3**
			24 h	16.8	15.0	0	2.6	7.4	*28.7*	**43.1**	**51.9**	**65.9**	**99.4**
Delattre 2012	Sepsis	Plasma	8 h	*20.6*	17.9	0	5.3	**44.8**	**49.1**	**57.3**	**54.3**	**87.1**	**97.3**
			24 h	*24.2*	*25.4*	0	2.6	15.4	**53.1**	**65.3**	**84.8**	**95.7**	**99.1**
		Lung	8 h	0	0	0	0	0	2.2	0	0	0	0
			24 h	0	0	0	0	0	8.5	0	0	0	0
Roberts 2009	Sepsis	Plasma	8 h	18.8	15.0	0	5.4	*30.2*	*35.9*	**47.5**	**45.0**	**94.9**	**99.0**
			24 h	19.3	16.5	0	1.8	10.1	*36.5*	**48.9**	**81.7**	**89.3**	**99.7**
		Lung	8 h	0	0	0	0	0	2.3	0	0	0	0
			24 h	0	0	0	0	0	7.1	0	0	0	0
Lu 2016	Neurosurgery	Plasma	8 h	12.7	0	0	4.6	15.4	*20.8*	*34.6*	0	6.5	*20.4*
			24 h	12.8	0	0	1.5	5.1	*20.8*	*34.7*	0	2.2	6.8
		Cerebrospinal fluid	8 h	0	0	0	0	0	0	0	0	0	0
			24 h	0	0	0	0	0	0	0	0	0	0
Wittau 2015	Obese	Plasma	8 h	14.2	0	0	0.4	19.4	*28.7*	*37.7*	0	0.4	*35.0*
			24 h	14.4	0	0	0.1	6.5	*28.7*	*38.3*	0	0.1	11.7
		s.c. interstitial space fluid	8 h	2.6	0	0	0	3.3	7.4	8.5	0	0	4.6
			24 h	8.0	0	0	0	3.3	*22.4*	*26.0*	0	0	4.6
		Peritoneal fluid	8 h	4.5	0	0	0	6.0	9.3	12.2	0	0	10.4
			24 h	13.5	0	0	0	6.0	*28.0*	*36.9*	0	0	10.4
Li 2006	Infected adults	CLCR 30 mL/min	8 h	*29.0*	*34.4*	0	*34.0*	**98.3**	**58.5**	**70.9**	**74.7**	**97.3**	**99.0**
			24 h	*32.5*	**40.5**	0	16.5	**48.6**	**61.7**	**76.3**	**91.5**	**99.1**	**99.7**
		CLCR 250 mL/min	8 h	8.3	0	0	3.1	9.2	14.2	*27.7*	0	3.3	10.4
			24 h	8.3	0	0	1.0	3.1	14.3	*28.1*	0	1.1	3.5
		EC_{50} 4.4 mg/L	8 h	**47.1**	**62.9**	**89.6**	**99.8**	**100**	**65.2**	**82.0**	**96.9**	**99.8**	**100**
			24 h	**47.5**	**64.0**	**96.5**	**99.9**	**100**	**65.8**	**83.1**	**99.0**	**99.9**	**99.8**
		EC_{50} 8.8 mg/L	8 h	*29.6*	**42.8**	0.7	**98.1**	**99.4**	**47.1**	**62.9**	**89.6**	**99.0**	**99.9**
			24 h	*30.2*	**43.9**	**66.9**	**99.3**	**99.7**	**47.5**	**64.0**	**96.5**	**99.7**	**99.9**
		EC_{50} 35.4 mg/L	8 h	5.6	0	0	0	6.2	14.6	12.2	0	0	6.7
			24 h	5.8	0	0	0	2.1	14.9	14.6	0	0	2.2
		k_{growth}, k_{death} -40%	8 h	14.6	12.2	0	6.7	*21.9*	*39.6*	**42.8**	0.7	**51.8**	**99.3**
			24 h	14.9	14.6	0	2.2	7.3	*30.2*	**43.9**	**66.9**	**82.9**	**98.1**
		k_{growth} -40%	8 h	14.6	12.2	0	6.7	*21.9*	*39.6*	**42.8**	0.7	**51.8**	**99.3**
			24 h	14.9	14.6	0	2.2	7.3	*30.2*	**43.9**	**66.9**	**82.9**	**98.1**
		Higher initial bact. load (S)	8 h	14.6	12.2	0	6.7	21.9	39.6	42.8	0.7	51.8	99.3
			24 h	14.9	14.6	0	2.2	7.3	30.2	43.9	66.9	82.9	98.1
		Higher initial bact. load (SR)	8 h	14.6	12.2	0	6.7	21.9	39.6	42.8	0.7	51.8	99.3
			24 h	14.9	14.6	0	2.2	7.3	30.2	43.9	66.9	82.9	98.1

Abbreviations: TDD: total daily dose; II: intermittent dosing; CI: continuous infusion; q8/24h: every 8/24 hours; LD: loading dose (in mg); CLCR: creatinine clearance; MIC: minimum inhibitory concentration; s.c. subcutaneous; EC_{50}: drug concentration (mg/L) that produces 50% of maximum achievable kill rate constant; kgrowth: rate constant of bacterial growth; kdeath: rate constant of natural bacterial death; higher initial bacterial load: 8 log10 CFU/mL, either assumed to be susceptible (S) or with an initial fraction in the resting state (SR); categories/colour code: $fT_{>MIC}$=95-100% (bold/underlined, mint), $fT_{>MIC}$=40-<95% (bold, light cyan), $fT_{>MIC}$=20-<40% (italic, beige), $fT_{>MIC}$=10-<20% (non-bold/non-italic, light red), $fT_{>MIC}$=<10% (non-bold/non-italic, violet red). Colours were intended to be accessible to people who are colour-blind (https://davidmathlogic.com/colorblind; access date 26.07.2022).

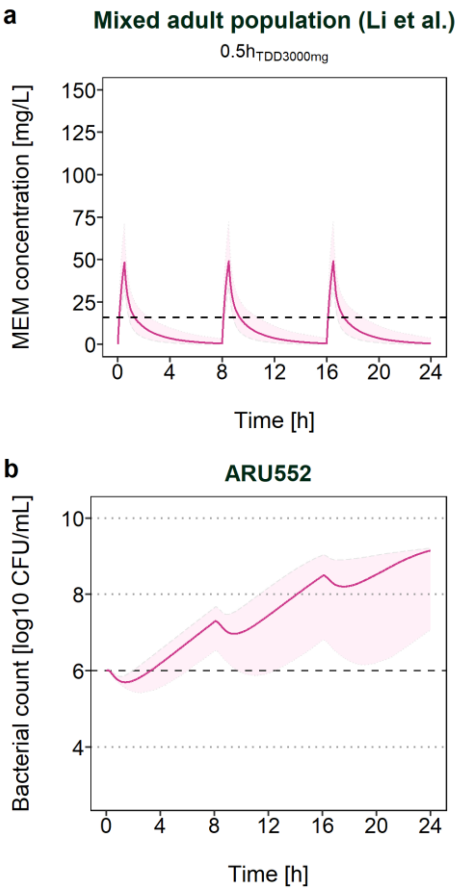

Figure S1. Concentration-time profile of meropenem (MEM, Figure a) and resulting bacterial load over time for a resistant *Pseudomonas aeruginosa* strain (ARU552, Figure b) given approved standard dosing of meropenem (1000 mg every 3 hours administered as 0.5-h infusions).

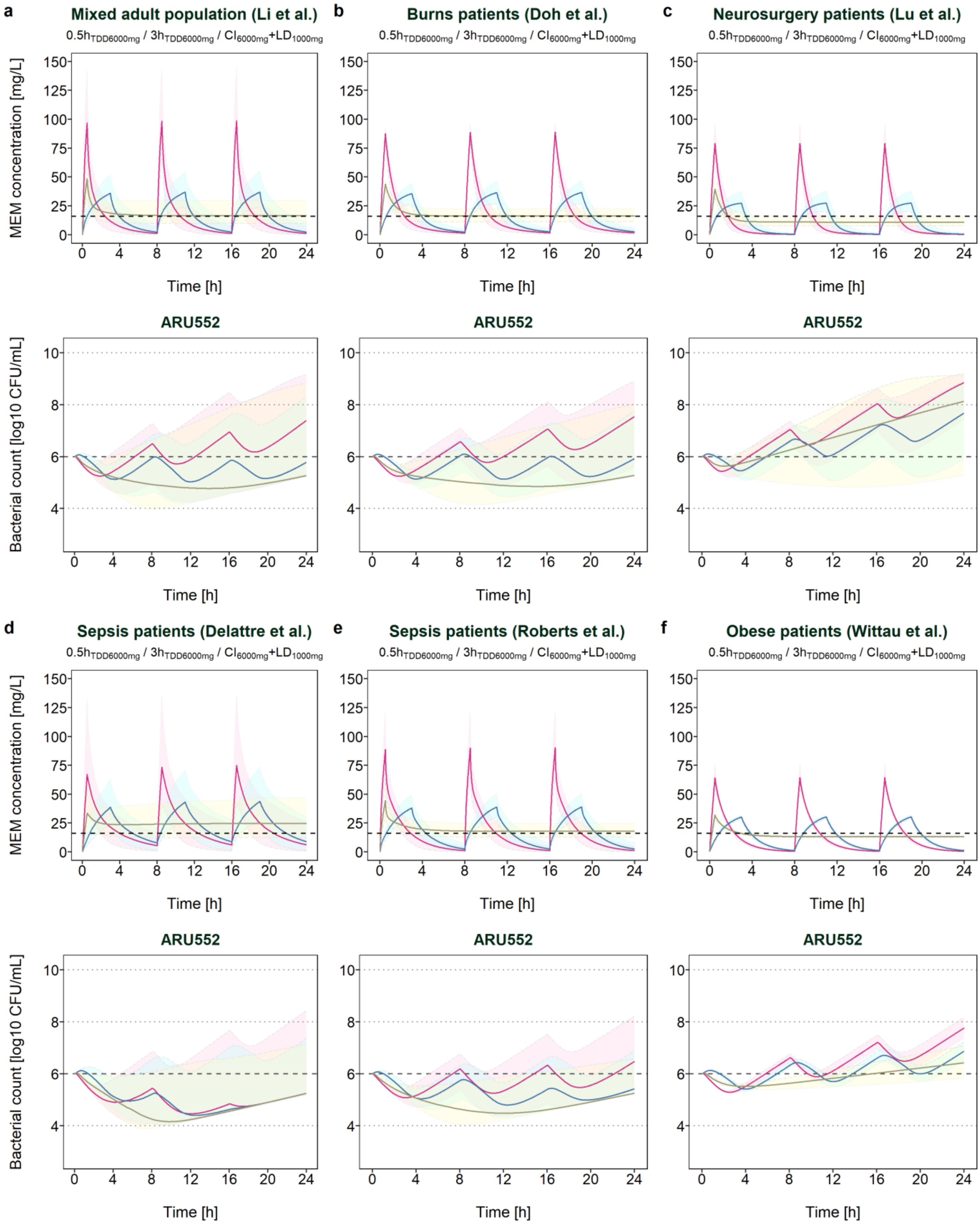

Figure S2. Concentration-time profiles of meropenem (MEM) and resulting bacterial load over time for a resistant *Pseudomonas aeruginosa* strain in six different patient populations (a-f) given three dosing regimens: (i) 2000 mg every 8 hours administered as 0.5-h infusions, (ii) 2000 mg every 8 hours administered as 3-h infusions, and (iii) 6000 mg/24 h administered as continuous infusion following a loading dose of 1000 mg.

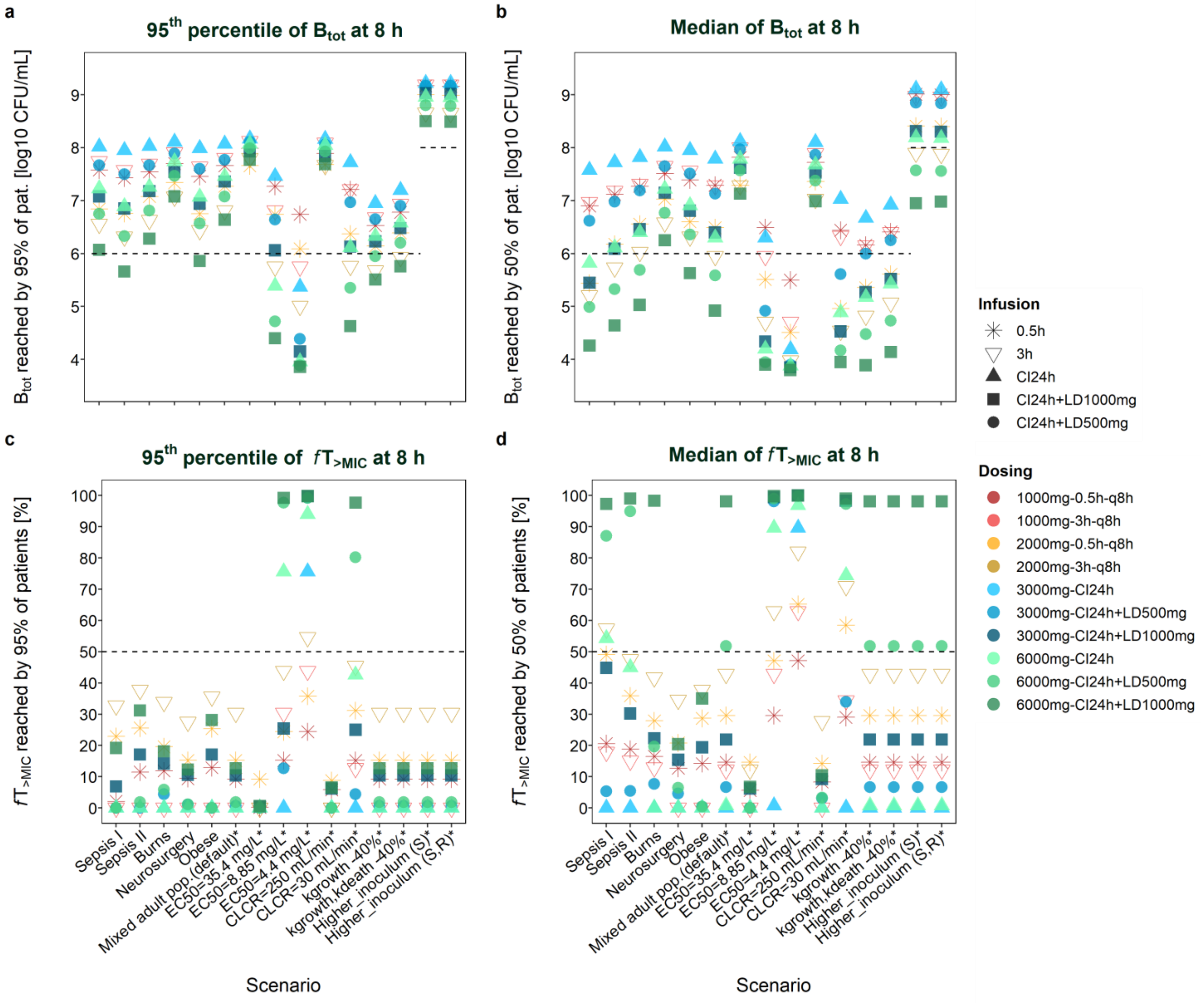

Figure S3. Total bacterial load (B_{tot}, Figures a and b) and $fT_{>MIC}$ (time that meropenem concentrations exceed the minimum inhibitory concentration, Figures c and d) reached by 95% (Figures a and c) or 50% (Figures b and d) of the patient (pat.) population **at 8 h** after start of therapy given different scenarios. Sepsis I: Delattre et al. [23]; Sepsis II: Roberts et al. [22] (no renal dysfunction); EC_{50}: drug concentration that produces 50% of E_{max} (maximum achievable kill rate constant); CLCR: creatinine clearance; kgrowth: rate constant of bacterial growth; kdeath: rate constant of natural bacterial death; higher inoculum: initial bacterial load of 8 log10 CFU/mL, either assumed to be susceptible or with an initial fraction in the resting state; scenarios marked with an asterisk * refer to a mixed adult population (default scenario; Li et al. [21]); CI: continuous infusion; LD: loading dose; q8h: every 8 hours.

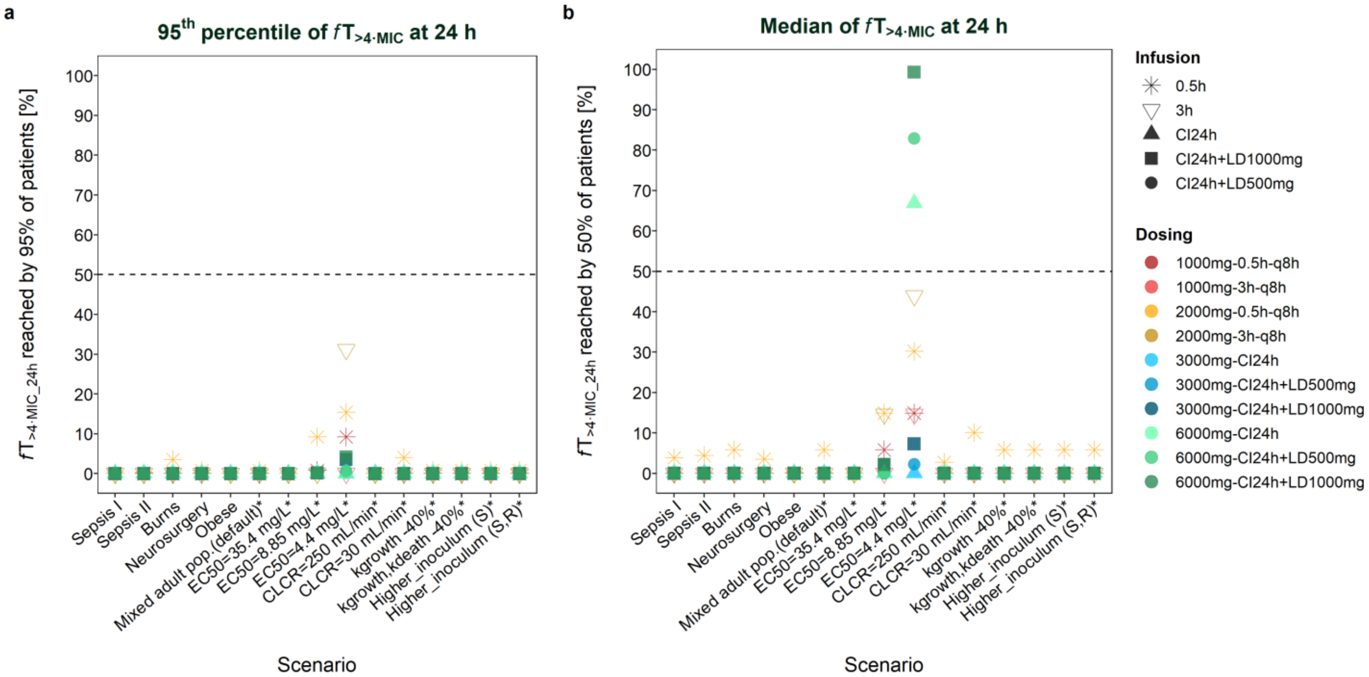

Figure S4. $fT_{>4 \cdot MIC}$ (time that meropenem concentrations exceed **four times the minimum inhibitory concentration**) reached by 95% (Figure a) or 50% (Figure b) of the patient population at 24 h after start of therapy given different scenarios. Sepsis I: Delattre et al. [23]; Sepsis II: Roberts et al. [22] (no renal dysfunction); EC_{50}: drug concentration that produces 50% of E_{max} (maximum achievable kill rate constant); CLCR: creatinine clearance; kgrowth: rate constant of bacterial growth; kdeath: rate constant of natural bacterial death; higher inoculum: initial bacterial load of 8 log10 CFU/mL, either assumed to be susceptible or with an initial fraction in the resting state; scenarios marked with an asterisk * refer to a mixed adult population (default scenario; Li et al. [21]); CI: continuous infusion; LD: loading dose; q8h: every 8 hours.

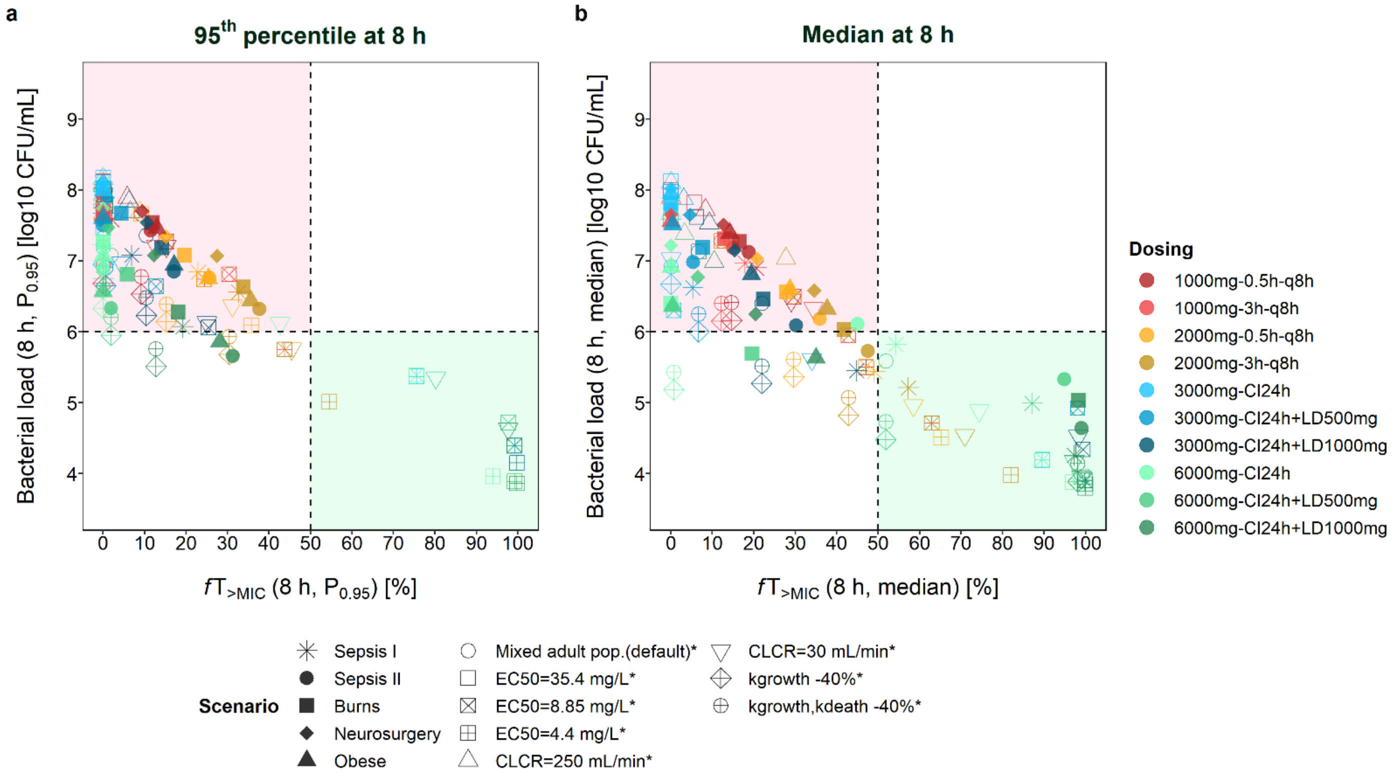

Figure S5. Total bacterial load versus $fT_{>MIC}$ (time that meropenem concentrations exceed the minimum inhibitory concentration) reached by 95% ($P_{0.95}$, Figure a) or 50% (median; Figure b) of the patient population **at 8 h** after start of therapy given different scenarios. Sepsis I: Delattre et al. [23]; Sepsis II: Roberts et al. [22] (no renal dysfunction); EC_{50}: drug concentration that produces 50% of E_{max} (maximum achievable kill rate constant); CLCR: creatinine clearance; kgrowth: rate constant of bacterial growth; kdeath: rate constant of natural bacterial death; higher inoculum: initial bacterial load of 8 log10 CFU/mL, either assumed to be susceptible or with an initial fraction in the resting state; scenarios marked with an asterisk * refer to a mixed adult population (default scenario; Li et al. [21]); CI: continuous infusion; LD: loading dose; q8h: every 8 hours.

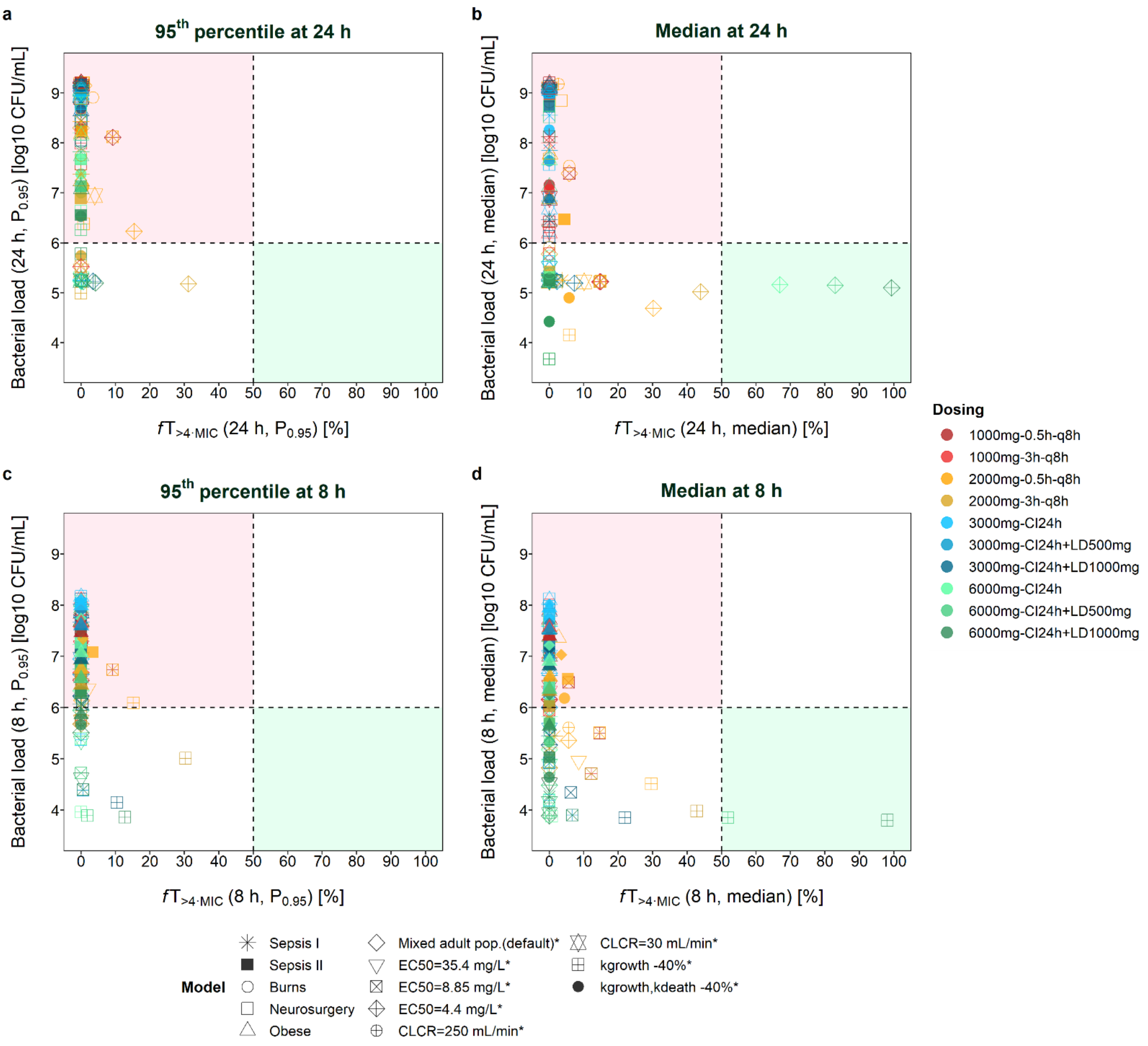

Figure S6. Total bacterial load versus $fT_{>4·MIC}$ **(time that meropenem concentrations exceed four times the minimum inhibitory concentration)** reached by 95% ($P_{0.95}$, Figures a and c) or 50% (median; Figures b and d) of the patient population at 24 h (Figures a and b) and 8 h (Figures c and d) after start of therapy given different scenarios. Sepsis I: Delattre et al. [23]; Sepsis II: Roberts et al. [22] (no renal dysfunction); EC_{50}: drug concentration that produces 50% of E_{max} (maximum achievable kill rate constant); CLCR: creatinine clearance; kgrowth: rate constant of bacterial growth; kdeath: rate constant of natural bacterial death; higher inoculum: initial bacterial load of 8 log10 CFU/mL, either assumed to be susceptible or with an initial fraction in the resting state; scenarios marked with an asterisk * refer to a mixed adult population (default scenario; Li et al. [21]); CI: continuous infusion; LD: loading dose; q8h: every 8 hours.
